# Mpox in Europe, 2022–2026: A Scoping Review of Viral Clades, Host Determinants of Severity and Antiviral Therapy

**DOI:** 10.3390/v18080865

**Published:** 2026-08-07

**Authors:** Filippos Sofos, Zoi D. Pana, Dimitris Drikakis

**Affiliations:** 1 Institute for Advanced Modelling and Simulation, University of Nicosia, Nicosia CY-2417, Cyprus; 2 Condensed Matter Physics Laboratory, Department of Physics, University of Thessaly, 35100 Lamia, Greece; 3 Department of Basic and Clinical Studies, University of Nicosia Medical School, Nicosia CY-2408, Cyprus

**Keywords:** mpox, Clade Ib, Clade IIb, tecovirimat, antiviral resistance, HIV co-infection, Europe

## Abstract

The 2022–2024 mpox outbreak in Europe was dominated by MPXV Clade IIb and generally mild, but recent Clade I/Ib detections and early local transmission have changed preparedness needs. We performed a PRISMA-ScR scoping review of European mpox evidence from 2022 to 2026, supplemented by global and African data where regional evidence was sparse, covering clades, host determinants of severe disease, and antiviral efficacy and resistance, and ranking the evidence gaps. European Clade IIb surveillance showed very low mortality (10 deaths among 22,662 cases; case fatality 0.04%), contrasting with 3–11% estimates for Clade I/Ib in affected African settings, although comparisons are confounded by age, health-system access and comorbidity. Severe European disease was driven mainly by immune compromise: non-HIV immunosuppression, uncontrolled HIV and CD4 counts < 200 cells/µL were the most consistent markers, while women were a small minority of cases, with higher hospitalisation risk, especially in pregnancy. Tecovirimat failed to improve outcomes in four randomised trials, F13L-associated resistance was reported in advanced HIV, and its European mpox use was subsequently restricted. The evidence base is strongest for mild Clade IIb infection in immunocompetent adults and weakest for the groups most likely to need treatment. Preparedness should prioritise harmonised genomic surveillance, severity-stratified cohort data and adaptive therapeutic trials enrolling immunocompromised, paediatric, pregnant and older patients.

## 1. Introduction

**Figure 1:**
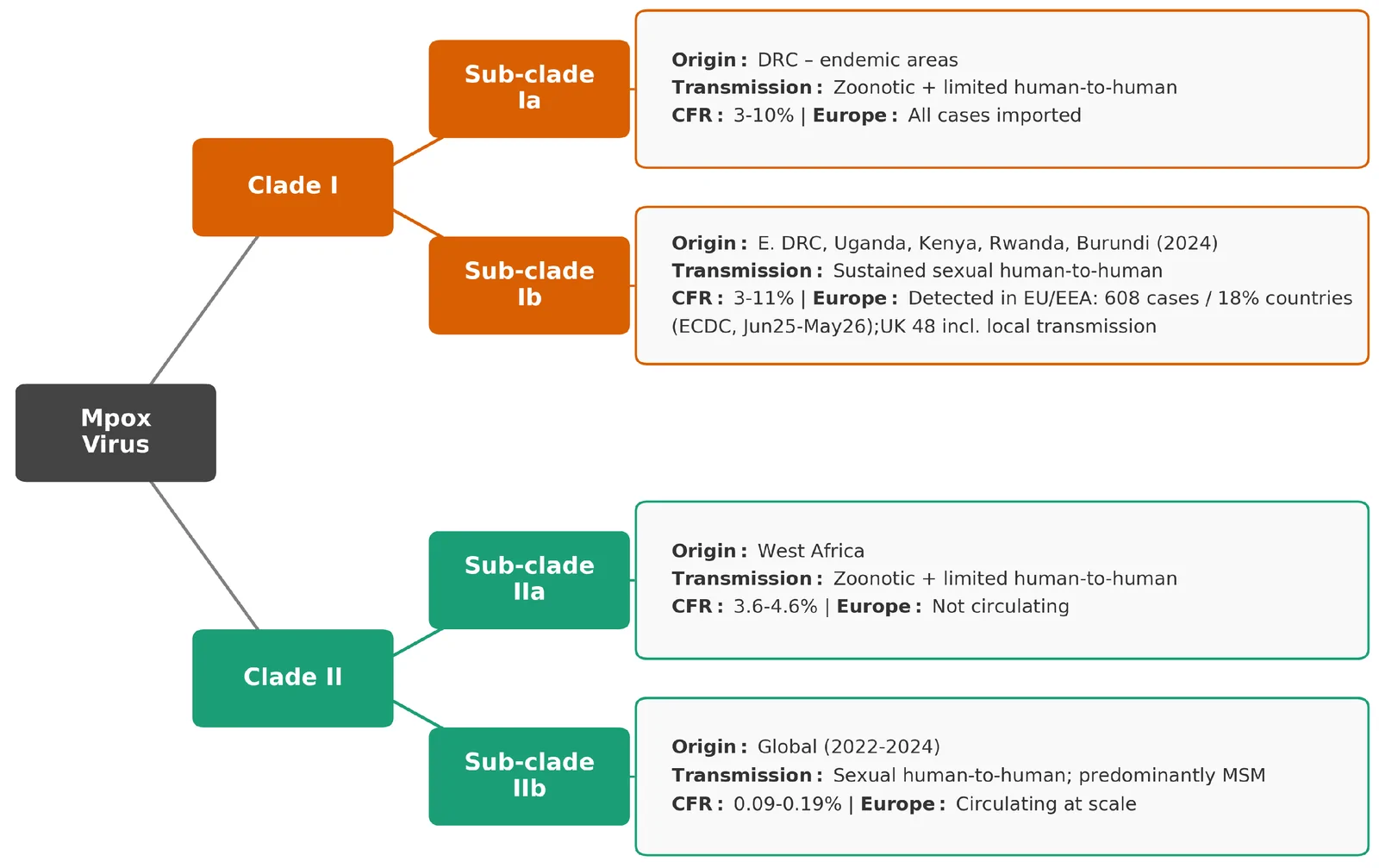
Epidemiological characteristics and geographical distribution of mpox clades.

Mpox is the clinical disease caused by the monkeypox virus (MPXV), a double-stranded DNA virus of the genus Orthopoxvirus that shares significant antigenic territory with the now-eradicated variola virus [1,2]. The virus was first identified in research macaques in 1958 and in a human patient in the Democratic Republic of the Congo (DRC) in 1970. For the next five decades, it behaved as a geographically circumscribed zoonosis, rarely passing through the forested regions of Central and West Africa, where transmission depended on close contact with animal reservoirs. Spillovers have been sporadic and onward transmission has been limited, mainly confined to households [1,3]. Two clades have been recognised: (i) Clade I (formerly Central African or Congo Basin) has been associated with case fatality rates of 3–10% and a heavy paediatric burden. It is further categorised in Clade Ia, traditionally endemic, characterised primarily by zoonotic transmission, and Clade Ib, which emerged in 2024, characterised by sustained human-to-human sexual transmission. (ii) Clade II (formerly West African) has given a milder course with lower lethality [3,4,5,6]. Here we have the sub-clades Clade IIa and Clade IIb. Clade IIb drove the 2022–2024 global outbreak and remains the only clade that has circulated at scale in Europe. Genotypic variations within the MPXV significantly influence both transmission efficiency and clinical outcomes. A detailed breakdown of these viral lineages, including their historical origins, transmission vectors, and European distribution, is summarised in the block diagram of Figure 1.

Routine smallpox vaccination ceased after variola eradication in 1980, and population-level orthopoxvirus immunity has declined steadily since then. That loss of cross-protective immunity is now widely regarded as the permissive substrate on which subsequent MPXV spread occurred, setting the stage for the global outbreak that emerged in 2022 [1,7,8].

The World Health Organisation (WHO) issued a Public Health Emergency of International Concern (PHEIC) in July 2022 [1,7,8], as an outbreak of MPXV, later attributed to Clade IIb lineage B.1, emerged almost in parallel across non-endemic countries in Europe, the Americas, and Asia within weeks. The global cumulative case count exceeded 87,000 in a year, while the European Union/European Economic Area (EU/EEA) had recorded more than 22,000 confirmed cases, with reporting across all 30 participating countries [7,8]. The demographic profile in EU was unlike any previous mpox episode, adult men who have sex with men (MSM) made up the great majority of cases, sexual transmission accounted for 94–96% of cases with ascertained exposure data, and HIV co-infection was documented in 38–52% [7,8,9,10]. Phylogenomic reconstruction placed the European epidemic within a monophyletic descendant of the 2017–2019 Nigerian outbreak strain (A.1), which carries an unusually high mutational load enriched for APOBEC3-compatible substitutions. This constituted molecular evidence of sustained, cryptic human-to-human circulation predating the recognised outbreak [11,12].

Nonetheless, the European Clade IIb picture was clinically reassuring. Fatality rate remained below 0.2% in national cohorts and 0.04% in pooled EU/EEA surveillance, with hospitalisation between 5% and 6% [7,8]. On the other hand, individual patient heterogeneity was considerable, as derived for the largest pan-European dataset, the six-country MOSAIC observational cohort (enrolling confirmed cases during 2022–2023). Most Clade IIb cases would not need special treatment, whereas the minority requiring systemic antivirals or hospitalisation was concentrated among patients with more extensive lesions and immunological compromise [13]. Cohort and surveillance work from Italy [10,14,15,16,17], Spain [18,19,20,21,22], Belgium [23,24], Germany [9], Denmark [25], Poland [26,27], the United Kingdom [28,29,30], and France [12] converge on a single dominant axis of severity: immunological status. Severe disease has been mainly linked to advanced HIV (CD4 < 200 cells/μL) and non-HIV immunosuppression. The roles of age extremes, female sex, and pregnancy have also been flagged, though the evidence supporting these associations remains less settled [8,10,21]. Traditional tracking systems mostly focused on MSM cases, which meant they missed other groups. However, when testing programs were set up outside of regular sexual health clinics, they managed to capture a small number of cases among women and heterosexuals [16,18,28], which further validates the hypothesis that the published European case profile is not a complete portrait of the underlying epidemic.

The overall picture has kept on changing. On 14 August 2024, the WHO declared the sudden rise of the Clade Ib variant a PHEIC, due to the ongoing human-to-human transmission through sexual contact in eastern DRC, alongside the rapid spread of the virus across borders into Burundi, Rwanda, Uganda, and Kenya [4,5,31]. On 15 August 2024, Sweden reported the first Clade Ib case detected within the EU/EEA [4,32]. By the end of June 2026, ECDC reported that since August 2024 several EU/EEA countries had recorded both travel-associated and locally-acquired mpox Clade I cases, with a new pattern of autochthonous transmission emerging among MSM in the EU/EEA [33]. The 24 October 2025 ECDC Threat Assessment Brief graded the risk of Clade Ib infection in the EU/EEA as moderate for MSM and low for the general population, while the risk of Clade IIb infection was assessed as low and very low, respectively [34]. In parallel, the UK Health Security Agency recorded confirmed Clade Ib cases up to 30 April 2026, with a documented contribution from secondary local transmission [35]. The clinical picture in Europe has shifted from preparing for a possible arrival of Clade Ib to responding to actual, low-volume Clade I/Ib transmission occurring alongside the residual Clade IIb activity that continues from the 2022 outbreak African surveillance continues to indicate preliminary Clade Ib case fatality rates in the 3–11% range (improving to 1.4–1.7% with structured supportive care), and a demographic profile dominated by children under 15 years, who account for around 70% of cases and approximately 85% of fatalities in current DRC data [3,4,5,31]. In Europe, such comparisons are not feasible at this stage. The European patient population now potentially at risk of Clade I exposure nonetheless differs systematically from the adult-MSM Clade IIb experience, and clinical pathways need to be sized accordingly.

A second shift is in the international emergency response architecture itself. On 5 September 2025, the WHO Director-General accepted the IHR Emergency Committee’s view that the 2024 mpox upsurge no longer met the criteria for a PHEIC, and the PHEIC was lifted accordingly [36]. Nevertheless, standing recommendations under the IHR (2005) were extended through August 2026, in recognition of continuing transmission risk and of asymmetric global capacity for surveillance and care [37]. The clinical and public-health bottom line for Europe is contradictory, since the international emergency guidance has been formally relaxed, while European Clade I/Ib transmission is beginning to be detected outside imported chains.

The antiviral landscape has narrowed in parallel. Tecovirimat (ST-246), an oral inhibitor of the orthopoxvirus VP37 envelope protein and the only small molecule developed specifically for orthopoxvirus disease, has entered European clinical practice through both regulatory and compassionate-use pathways on the strength of preclinical and animal-model data, with very little randomised human mpox evidence [1,2,3,38]. The pivotal randomised evidence then delivered convergent negative results across four trials, one in Clade I disease and three in Clade II. PALM007 (NIAID, DRC) enrolled approximately 597 patients with Clade I mpox and found no significant effect on time to lesion resolution or mortality [39]. In Clade II disease, the NIH-sponsored STOMP trial in a predominantly immunocompetent international population [40], the ANRS/MPX-Response UNITY trial (Argentina, Brazil and Switzerland) [41], and the UK PLATINUM-UK trial [42] each likewise failed to meet the primary endpoint on time to lesion resolution (or scabbing, for PLATINUM-UK). Therefore, two of the three Clade II trials contributed European patient data (UNITY through the Swiss site and PLATINUM-UK from the UK).

Reports of F13L-mediated tecovirimat resistance in patients with advanced HIV on prolonged monotherapy [43,44] increased the clinical concern, and the European Medicines Agency restricted tecovirimat’s use for mpox in March 2026 [45]. Second-line agents, cidofovir and brincidofovir (both targeting the viral DNA polymerase E9L) and Vaccinia Immune Globulin (VIGIV), remain available only through compassionate-use channels and lack mpox-specific randomised data [1,2,3,38]. The patient groups in whom an antiviral effect would be most clinically meaningful, such as those with advanced HIV, non-HIV immunosuppression, paediatric Clade I exposure, pregnancy, and old age, remain untested.

Given the rapid changes in surveillance and treatment evidence, it is crucial that we bring together all the European data to get a clear picture of the current landscape. The published European literature describes a mild, demographically narrow Clade IIb experience. Early Clade I/Ib transmission is now being layered onto this residual Clade IIb circulation in the EU/EEA, but with a very different clinical and demographic profile, as antiviral options narrow in the face of negative trial results and an emerging resistance signal. The contribution of this scoping review is to bring these strands together, i.e., the European Clade IIb clinical and genomic experience, the new risk of Clade Ib importation, and the fast-changing antiviral evidence, in order to highlight a practical mismatch that earlier broad reviews have not. Most of the evidence covers low-risk, largely immunocompetent Clade IIb patients, whereas the groups most likely to need treatment, such as advanced HIV, non-HIV immunosuppression, pregnancy, children and older adults, are the least studied.

To address this gap, we conducted a scoping review of the published European mpox literature along three axes: clades, population demographics, and antivirals, drawing on global and African evidence where European data are sparse. The review was built on the cohort projects coordinated through the Cohort Coordination Board (CCB) Central [46], complemented by bibliographic database and citation searches. Full methodological details are given in Section 2 and the Supplementary Materials. Next, we present all relevant reviews and meta-analyses from recent years that report European mpox trends (Section 3). We map the clade and lineage makeup of the European outbreak and compare intra-European Clade IIb severity with Clade I/Ib data from Africa and early European chains (Section 4). We then derive a hierarchy of demographic and immunological predictors of severe mpox in European populations (Section 5) and appraise the evidence for the safety, efficacy, and resistance of tecovirimat, cidofovir, brincidofovir, and VIGIV in European practice (Section 6). Finally, we integrate the remaining evidence gaps into a prioritised research agenda for European mpox preparedness and clinical care (Section 7).

## 2. Materials and Methods

**Figure 2:**
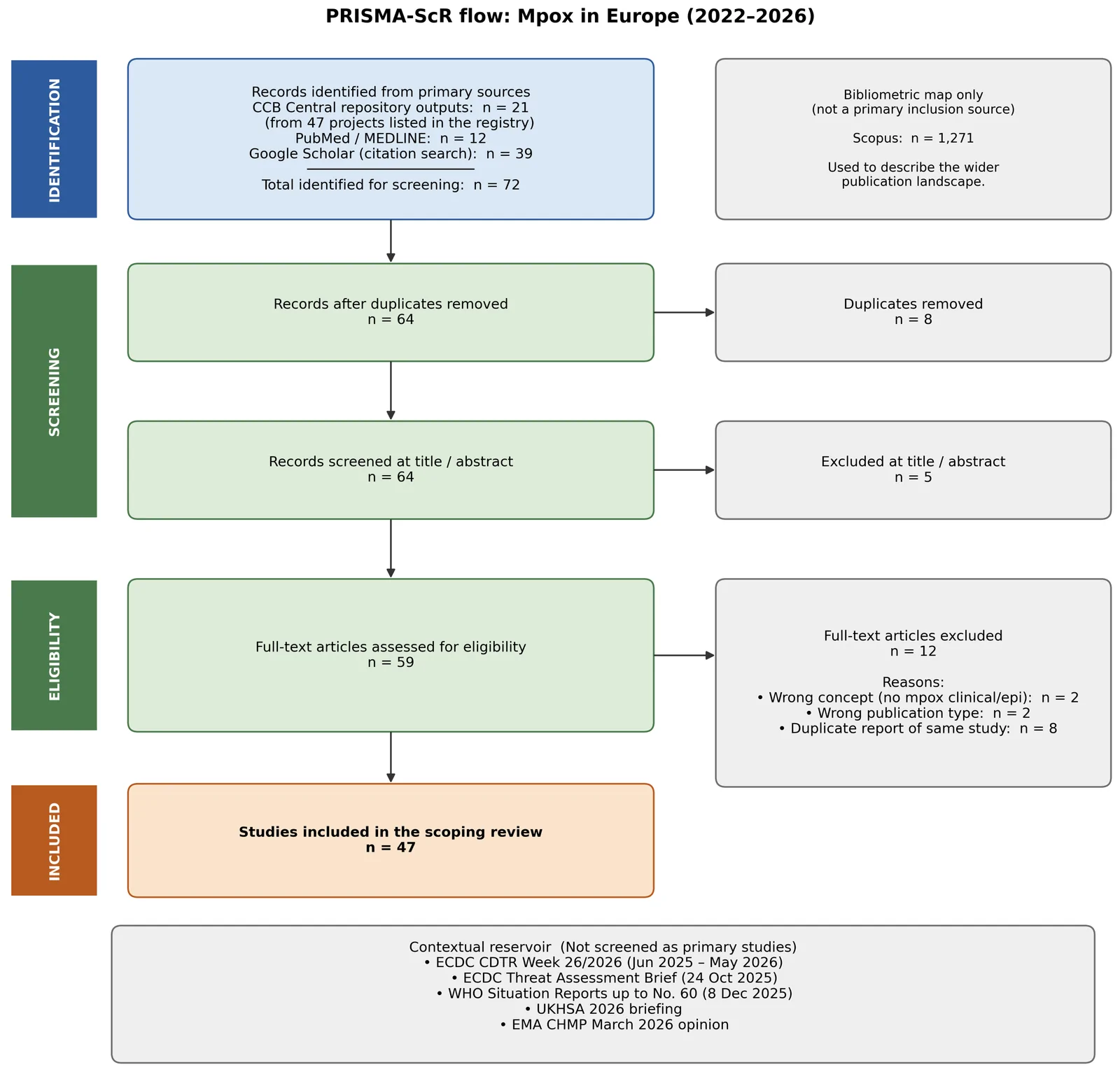
PRISMA-ScR flow of records. CCB Central repository (registry) and PubMed/MEDLINE with evidence-synthesis and trial filters were the primary inclusion sources, Google Scholar provided citation-search coverage, while Scopus (1271 records) served as a bibliometric map of the wider field. Contextual surveillance/regulatory documents (ECDC, UKHSA, WHO, EMA) were used for epidemiological framing only.

This scoping review was designed, conducted, and reported in accordance with the Preferred Reporting Items for Systematic reviews and Meta-Analyses extension for Scoping Reviews (PRISMA-ScR) [47]. Figure 2 presents the flow of records through identification, screening and inclusion. Database and registry counts are drawn from the search logs and screening counts follow the review’s records. A completed PRISMA-ScR checklist mapping every one of the 22 reporting items to its manuscript location, together with the review protocol, the full search strategies, the Population–Concept–Context (PCC) eligibility framework, the data-charting form, and the evidence-gap scoring rubric are provided in the Supplementary Materials. The underlying methodological guidance follows the JBI Manual for Evidence Synthesis on scoping reviews [48]. The review was not registered because the International Prospective Register of Systematic Reviews (PROSPERO) [49] does not currently accept scoping review protocols.

The main literature window was 1 January 2022 to 30 April 2026, and the final search across all information sources was executed again on 30 June 2026, updating some of the results presented, if there was a new addition. We note that not all formal EU reports or cohort data are updated at the same time, so results presented here do not always follow the same time window. Four sources were used: (i) the CCB Central Data Repository as the primary registry, from which 47 European mpox projects were identified and 21 publications and outputs retrieved [46], (ii) PubMed/MEDLINE restricted to evidence synthesis and randomised trial article types, from which 12 records were identified, (iii) Google Scholar for backward and forward citation searching, from which 39 papers were retrieved, and (iv) Scopus (1271 records) as a bibliometric map of the wider field. Full search strings and filters for each source are given in Supplementary Section 5. Surveillance and regulatory documents released after the search close (ECDC CDTR Week 26/2026, WHO Situation Report No. 60, UKHSA 2026 briefing, EMA CHMP March 2026 opinion) were retained as contextual reservoirs for epidemiological framing only and did not undergo the same screening process as primary studies.

Records were de-duplicated by DOI and screened in two stages (title/abstract, then full text) against the PCC criteria, with dual independent review and consensus arbitration. The included set was charted into a pre-specified 14-variable form (Supplementary Section 7) and synthesised narratively and in tables along three axes, i.e., clades, host determinants of severity, and antivirals. Because included evidence spans heterogeneous designs (surveillance bulletins, prospective and retrospective cohorts, randomised trials, case series, and regulatory documents), no pooled effect estimate was computed. In line with the PRISMA-ScR framework and JBI guidance for scoping reviews, no formal risk-of-bias appraisal was undertaken [47].

## 3. Recent Perspectives on Mpox in Europe

Over the past year, updated clinical guidelines, meta-analyses, and systematic reviews have offered a much more precise retrospective assessment at the 2022–2025 mpox outbreak, bringing Europe’s unique regional patterns into focus (see Table 1). When examined together, they reveal that while the disease was less severe in Europe than the global average, the outbreak still exposed real flaws in how healthcare facilities prepared and how research was coordinated. As the landscape is still shifting and new post-outbreak data has recently emerged, we have limited the scope of our review to literature published strictly within the last year. Focusing solely on this recent timeframe allows us to refresh the current knowledge base with refined epidemiological data and updated guidelines informed by long-term European outcomes.

A recent global review places Clade IIb wave in Europe within the wider 2022–2024 timeline and warns that the virus has the capacity for further evolution [55]. Meta-analyses consistently rank Europe lowest worldwide for mpox severity. For instance, the pooled case fatality ratio (CFR) was 0.1% in Europe versus 3.1% globally [51], and hospitalisation rates were the lowest of any region, in contrast to endemic African settings [52]. Despite these mild aggregate outcomes, the European AIDS Clinical Society (EACS) guidelines (v13.0) revised mpox co-infection and tecovirimat guidance for vulnerable groups, particularly people with HIV [50].

Nevertheless, the outbreak strained European health and research systems. A systematic review found recurrent facility-level challenges, including space limitations, task-shifting, and staff mental-health strain [54], while an evaluation of 49 European mpox cohorts reported heterogeneous data collection that hindered cross-border pooling, prompting calls for standardised, ever-warm cohorts maintained between outbreaks [53]. Continued detections, including among PrEP users in Croatia [56], reinforce the need for sustained surveillance in sexual health settings even as incidence falls. Finally, initiatives such as Project ODIN aim to align African genomic surveillance with European preparedness against Clade I incursion [57].

**Figure 3:**
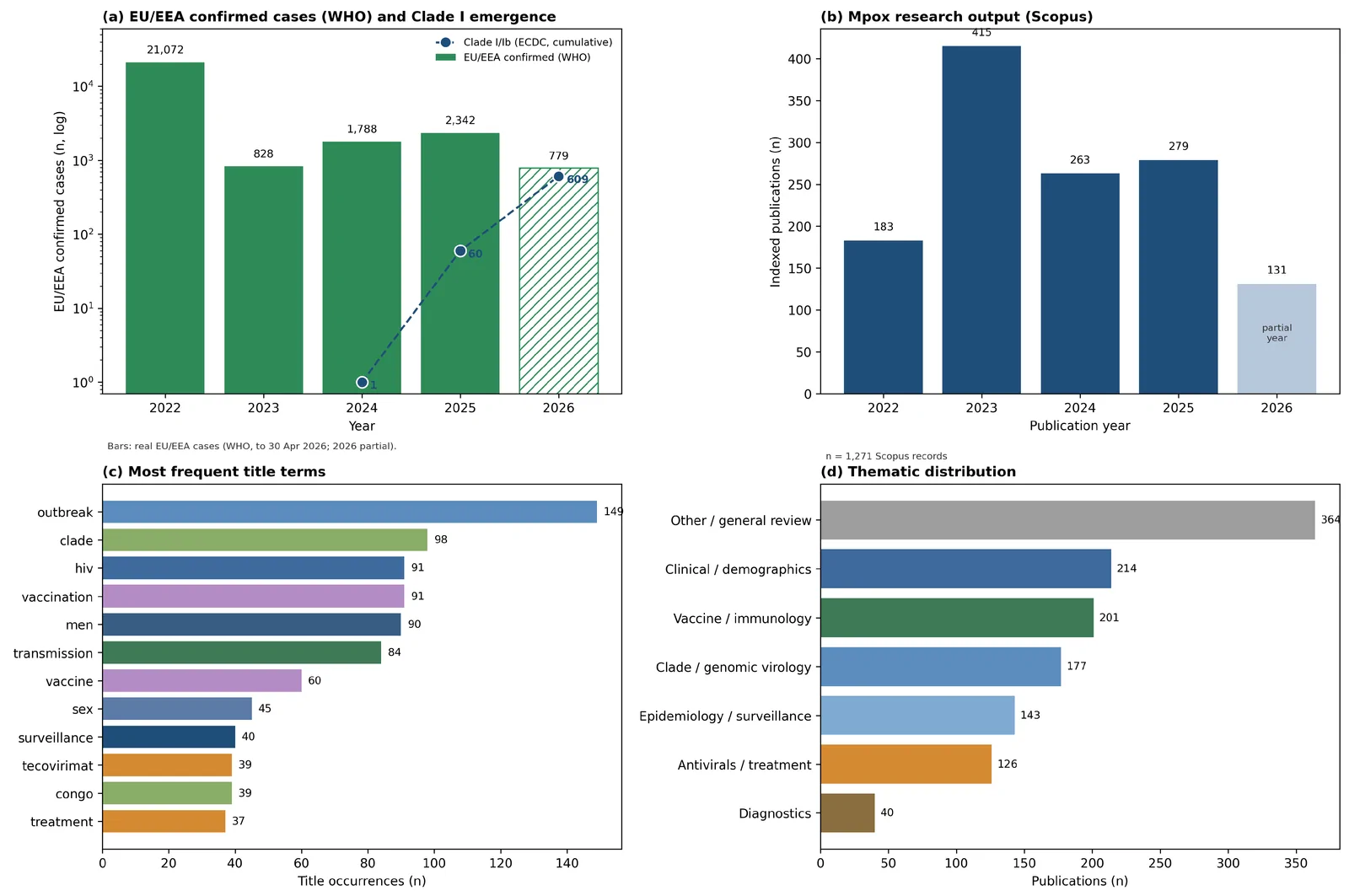
Mpox research landscape and EU/EEA surveillance context. (**a**) EU/EEA confirmed cases per year by clade (ECDC/WHO/UKHSA–2026 partial). (**b**) Indexed mpox publications per year from Scopus (*n* = 1271, 2022–2026, 2026 partial). (**c**) Most frequent title terms. (**d**) Thematic distribution by title keyword classification. Panels (**b**–**d**) come from the Scopus export and (**a**) from European surveillance.

Over the same window, EU/EEA surveillance recorded 21,072 Clade IIb cases in 2022, 828 in 2023, 1789 in 2024 and 2342 in 2025, indicating continued transmission. Superimposed on this residual Clade IIb circulation, Clade I/Ib expanded from a single imported EU/EEA case in August 2024 to 608 confirmed cases across 18 countries during June 2025–May 2026 [33], with 48 further cases in the UK to 30 April 2026 [35]. This pattern, with Clade I/Ib now emerging inside the EU/EEA even as Clade IIb continues at low levels, is the new epidemiological shift that motivates this review (Figure 3a).

A Scopus export of 1271 records (2022–2026) maps the wider research landscape (Figure 3b). From 183 records in 2022, we reached a peak of 415 in 2023 (i.e., a year after the case peak, an outbreak-to-publication lag), followed by a decreased plateau in 2024 and 2025. The most frequent title terms track the outbreak’s phenotype, with *outbreak* (149), *clade* (98), *vaccination*/*vaccine* (91/60) and the HIV-associated MSM signature (*hiv* 91, *men* 90, *sex* 45), whereas *tecovirimat* (39) and *treatment* (37) are rare (Figure 3c). Thematically, reference output concentrates on clinical and demographic characterisation (*n* = 214), vaccine immunology (*n* = 201), clade or genomic virology (*n* = 177) and epidemiology/surveillance (*n* = 143), whereas the two most treatment-facing themes are the least represented, with antivirals/treatment at *n* = 126 and diagnostics the lowest at *n* = 40 (Figure 3d). Thus, the evidence base is rich in prevention and in the characterisation of mild Clade IIb disease, but poor in the diagnostic and therapeutic questions most relevant to severe, high-risk patients.

## 4. The European Outbreak of MPXV

### 4.1. Clades Identified in Europe

**Figure 4:**
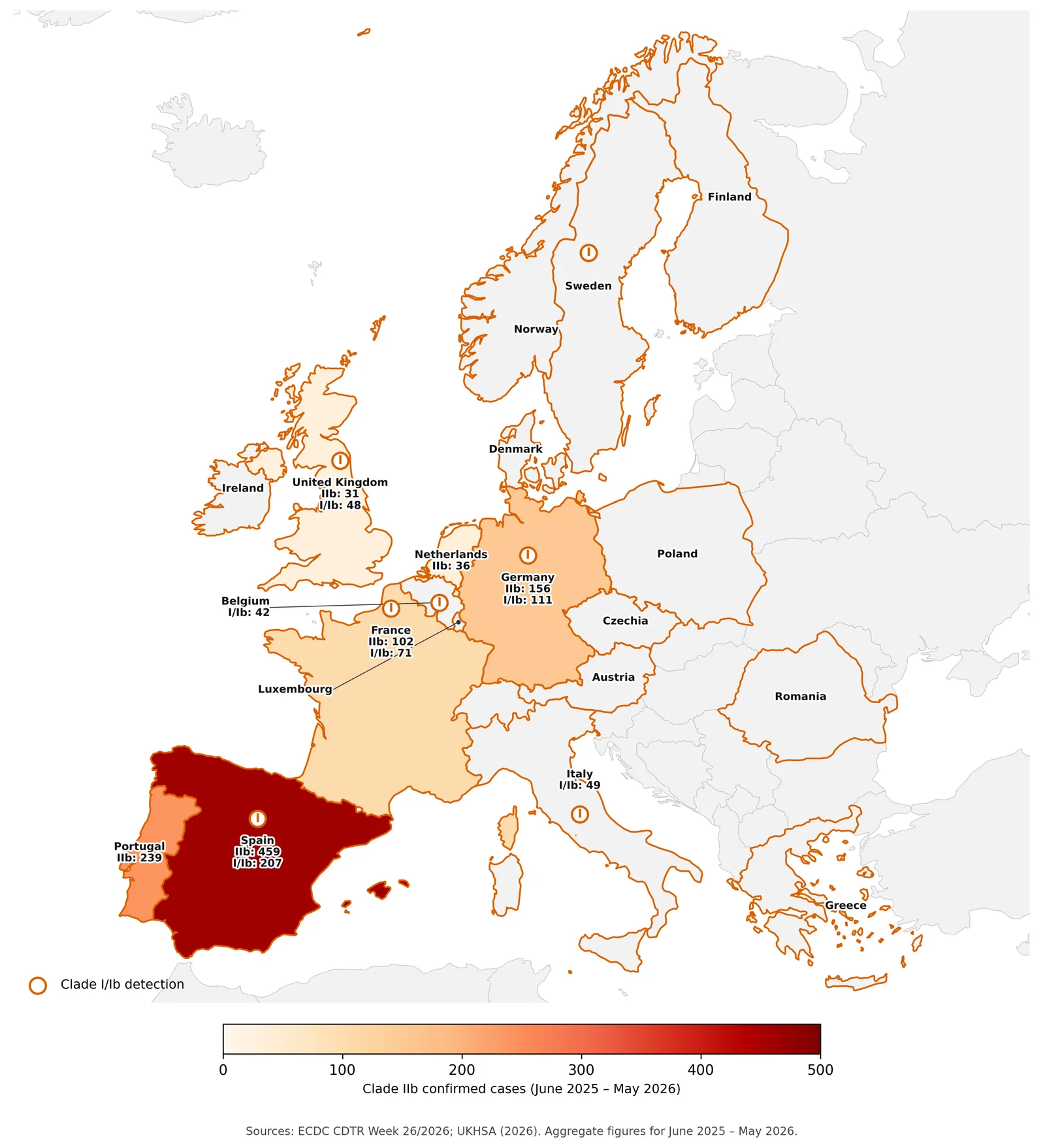
Geographic distribution of MPXV in Europe: 18 EU/EEA countries with confirmed Clade I detection during June 2025–May 2026, plus the UK. The colour scale shows the cumulative number of reported Clade IIb cases during Period 2 (2023–2024); hatched fill indicates countries without a Clade IIb Period-2 count in the reviewed corpus. Both Clade IIb cases (2023–2024) and Clade I cases (June 2025–May 2026) are shown. The marker “I: +” denotes confirmed Clade I detection without an individual published count. Boxes with orange outline, marked with a circled “I” denote all EU/EEA countries and the UK with confirmed Clade I detection in the review window. Sweden is kept as the imported EU/EEA index case (August 2024) [33].

The 2022–2024 mpox epidemic in Europe was driven almost exclusively by MPXV Clade IIb, a sub-clade historically associated with West African zoonotic transmission that acquired efficient human-to-human, sexually transmitted transmission during the global outbreak. The geographic distribution across European countries is shown in Figure 4 and analysed in Table 2. Surveillance data from the EU/EEA confirm [7]:

- 97% (*n* = 1943 sequenced cases) during the 2022–2024 outbreak window were Clade IIb, 13 were Clade IIa.
- Following the Swedish August 2024 index case, 18 EU/EEA countries reported 608 confirmed Clade I cases during June 2025–May 2026, initially travel-associated and subsequently including autochthonous transmission chains among MSM [33,35].
- Dominant Clade IIb lineage: B.1 (Period 1, 2022). Period 2 (2023–2024) showed emergence of distinct B.1 sub-clusters and the C.1 lineage. Low-level Clade IIb circulation has persisted alongside emerging Clade I transmission in 2025–2026.

Phylogenomic reconstruction placed the predominant European lineage within hMPXV-1 Clade B.1, a monophyletic group descended from the 2017–2019 Nigerian outbreak strain (A.1) and carrying an unusually high mutational load [11]. The substitutions were strongly enriched in GA→AA and TC→TT transitions, the signature of host APOBEC3 cytidine deaminases, indicating sustained, cryptic human-to-human circulation predating the recognised outbreak and providing the molecular basis for reclassifying the West African clade as Clade IIb, lineage B.1 [11].

After the May 2022 index detections, B.1 spread rapidly across non-endemic countries, with phylogeographic analyses inferring multiple independent introductions into individual European jurisdictions [11]. Deep genomic characterisation of 148 isolates from the Paris area corroborated this, identifying several co-circulating B.1 sub-lineages incompatible with a single source case and instead consistent with repeated importations into densely connected sexual networks. It also documented additional APOBEC3-compatible sequences that arose during the outbreak, providing evidence of continued host-driven micro-evolution [12].

Three implications follow. First, the near-monoclonal Clade IIb composition of European cases precludes within-region inference on clade-specific virulence, so severity comparisons across Clades Ia, Ib and IIb must rely on cross-continental data. Second, because B.1 was established through repeated, dispersed introductions, control required coordinated, network-level measures rather than single-chain containment. Third, the ongoing accumulation of APOBEC3-mediated mutations highlights the need for prospective genomic surveillance of residual or re-emergent Clade IIb cases and imported Clade Ib cases to detect phenotypically consequential divergence early [11,12].

The global mpox landscape changed on 14 August 2024, when WHO declared the Clade Ib upsurge a PHEIC in response to sustained sexual transmission in eastern DRC and cross-border spread. DRC, Uganda, Madagascar, Burundi and Kenya have reported the largest Clade I case counts since 2025, and while African Clade I cases have declined overall since May 2025, Madagascar sustained transmission through May 2026 [4,5,31,33]. The following day, Sweden reported Europe’s first imported Clade Ib case in a returning traveller [4,32]. Whereas early European detections were travel-associated, ECDC has since reported 608 confirmed Clade I cases from 18 EU/EEA countries during June 2025–May 2026 [33], and UKHSA recorded 48 Clade Ib cases to 30 April 2026, including documented secondary local transmission [35]. The situation in Europe has shifted from a hypothetical threat to a real one. We are now seeing the early, small-scale spread of Clade I/Ib, along with residual Clade IIb cases.

On 5 September 2025 WHO determined that the 2024 upsurge no longer met PHEIC criteria and lifted it [36], while Standing Recommendations under the IHR (2005) were extended to August 2026 given continuing transmission risk [37].

### 4.2. Association of Clades with Clinical Outcomes and CFR

As Clade IIb has been the only MPXV clade to circulate at scale in Europe, and the recent Clade I/Ib detections remain small for meaningful statistical analysis [32,35], direct intra-European comparisons of CFR and clinical severity across clades are not yet feasible. Therefore, inter-clade comparisons rely on triangulating European data on Clade IIb with African surveillance for Clades Ia and Ib and with imported case series. The main parameters relevant to a clinical and public health comparison are summarised in Table 3.

A critical observation is the approximately hundred-fold difference in CFR between Clade I and Clade IIb. In European Clade IIb cohorts, the CFR is always below 0.2%, and the absolute number of deaths recorded in the EU/EEA during the 2022–2024 outbreak (10 deaths among 22,662 confirmed cases, 0.04%) is an order of magnitude lower than even the lower bound of published Clade Ia estimates [4,5,31]. Conversely, African surveillance of Clade Ib epidemic in the eastern DRC and neighbouring countries has reported preliminary CFRs in the 3–11% range, with marked improvement (1.4–1.7%) when patients are managed under structured supportive care [4,5,31].

Several caveats are essential to the correct interpretation of these figures. First, CFR estimates for Clade I derive from settings with limited diagnostic capacity, restricted access to hospital-level support and high background prevalence of malnutrition, untreated HIV and concurrent endemic infections, all of which increase mortality from any severe viral illness [2,3,4,6]. In contrast, the European Clade IIb estimate derives from a near-universal-access health system context with rapid diagnostic confirmation, linkage to sexual health services, and prompt antiviral or supportive interventions where required. Second, the demographic structure of the two epidemics differs significantly, as the European Clade IIb outbreak is concentrated in adult MSM, with paediatric cases being less than 1% [7,8,9], whereas DRC Clade I affect children under 15 years at most, who account for approximately 70% of cases and upwards of 85% of deaths in 2024 data [4,5,31]. In such a way, direct clade-attributable virulence cannot be inferred from CFR comparison without adjustment for age, baseline health status, healthcare access and HIV co-infection.

Third, the severity of mucocutaneous disease in European Clade IIb cases has been limited and self-resolving, although sparse presentations have been documented in immunocompromised individuals, in particular those with advanced, untreated HIV infection [3,8,10,13,14]. Reports from African Clade I cohorts suggest more extensive cutaneous and mucosal involvement, but heterogeneity in case definitions and case ascertainment again limits direct comparison. Therefore, the available evidence supports a biological gradient in virulence between Clade I and Clade IIb, significantly amplified by differences in host and healthcare setting. The clinically relevant message for European practice is that Clade Ib cases should not be assumed to follow the mild trajectory of Clade IIb, even when managed in well-resourced settings, and warrant heightened clinical vigilance, proactive supportive care and active consideration of antiviral therapy.

Within Clade IIb, no consistent association between specific viral lineage (B.1 versus the more recently described C.1 sub-cluster) and clinical severity has been found in the relevant literature. The C.1 lineage emerged during the 2023–2024 phase of the outbreak, but the epidemiological and clinical profiles of cases attributed to C.1 have been indistinguishable from those of contemporaneous B.1 cases [7,27].

The most informative dataset available is the EU/EEA phylogenetic and epidemiological assessment by Vaughan and colleagues, who stratified surveillance data into Period 1 (2022, predominantly B.1) and Period 2 (2023–2024, including emerging B.1 sub-clusters and the C.1 lineage) [7]. Hospitalisation rates were comparable across the two periods (5.3% in Period 1 versus 6.0% in Period 2), and absolute death counts remained low in both intervals (7 in Period 1/3 in Period 2), with no statistically significant lineage-associated difference in either outcome [7]. An atypical case of Clade IIb lineage C.1 reported from Poland by Zmora and colleagues (a 25-year-old MSM with HIV co-infection and a preserved CD4 count of 568 cells/μL who presented with widespread vesiculopustular lesions and mucous membrane involvement) illustrates that severe presentations can occur within Clade IIb, but appears more plausibly explained by host immunological factors than by an intrinsic lineage effect [27].

However, a clear evidence gap remains. To our knowledge, no European study has performed a formal multivariable comparison of clinical outcomes (severity scores, hospitalisation, mortality, duration of viral shedding) between Clade IIb B.1 and C.1 lineages with adjustment for age, HIV status, immunosuppression and access to antiviral therapy. Given the continued low-level circulation of Clade IIb in several European countries and the documented ongoing accumulation of APOBEC3-mediated mutations, prospective genomically informed cohort studies remain warranted to detect any phenotypic divergence at the earliest opportunity.

## 5. Population Demographics as Predictors of Outcome

### 5.1. Sex and Clinical Outcomes

**Figure 5:**
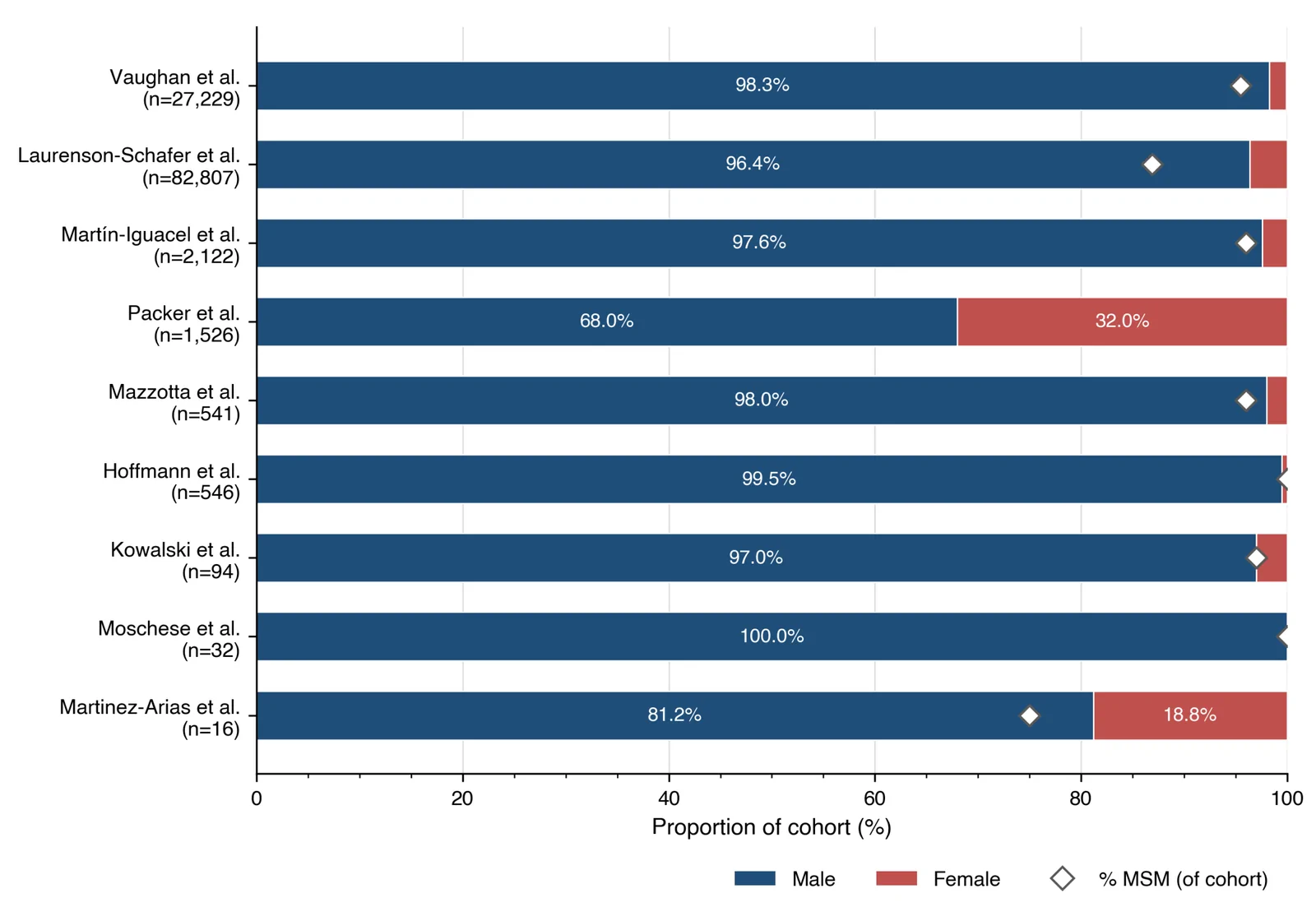
Sex composition and MSM proportion across European and global mpox cohorts, for males (blue bars) and females (red bars) per cohort. The diamond marker indicates the proportion of cases identifying as MSM [7,8,9,10,14,18,21,26,28].

The 2022–2024 European mpox outbreak was demographically distinct from all previous mpox epidemics, mainly concentrated in adult men, especially MSM. Across the surveillance, hospital and primary care cohorts reviewed here (Figure 5), males comprised between 96% and 100% of confirmed cases, with the EU/EEA surveillance system reporting 98.3% male and 95.5% MSM identification among those with completed exposure data [7]. The only cohorts in which the female proportion materially exceeded 5% were the UK household-contact study, in which non-sexual intra-household exposure produced a 32% female case fraction [28], and the Spanish primary care detection programme of Martínez-Arias and colleagues (18.8% female), in which screening outside specialist sexual health services revealed a small but clinically distinct heterosexual and non-MSM associated transmission pattern that would otherwise have remained undetected [18]. Consistent with this, Catalano and colleagues found mpox prevalence of only 0.53% among 189 cisgender women attending a high-risk STI clinic in Milan, despite intensive sexual health screening [16], reinforcing the conclusion that the European Clade IIb outbreak generated a very small absolute female case burden but that targeted surveillance outside MSM networks remains important for case ascertainment.

**Figure 6:**
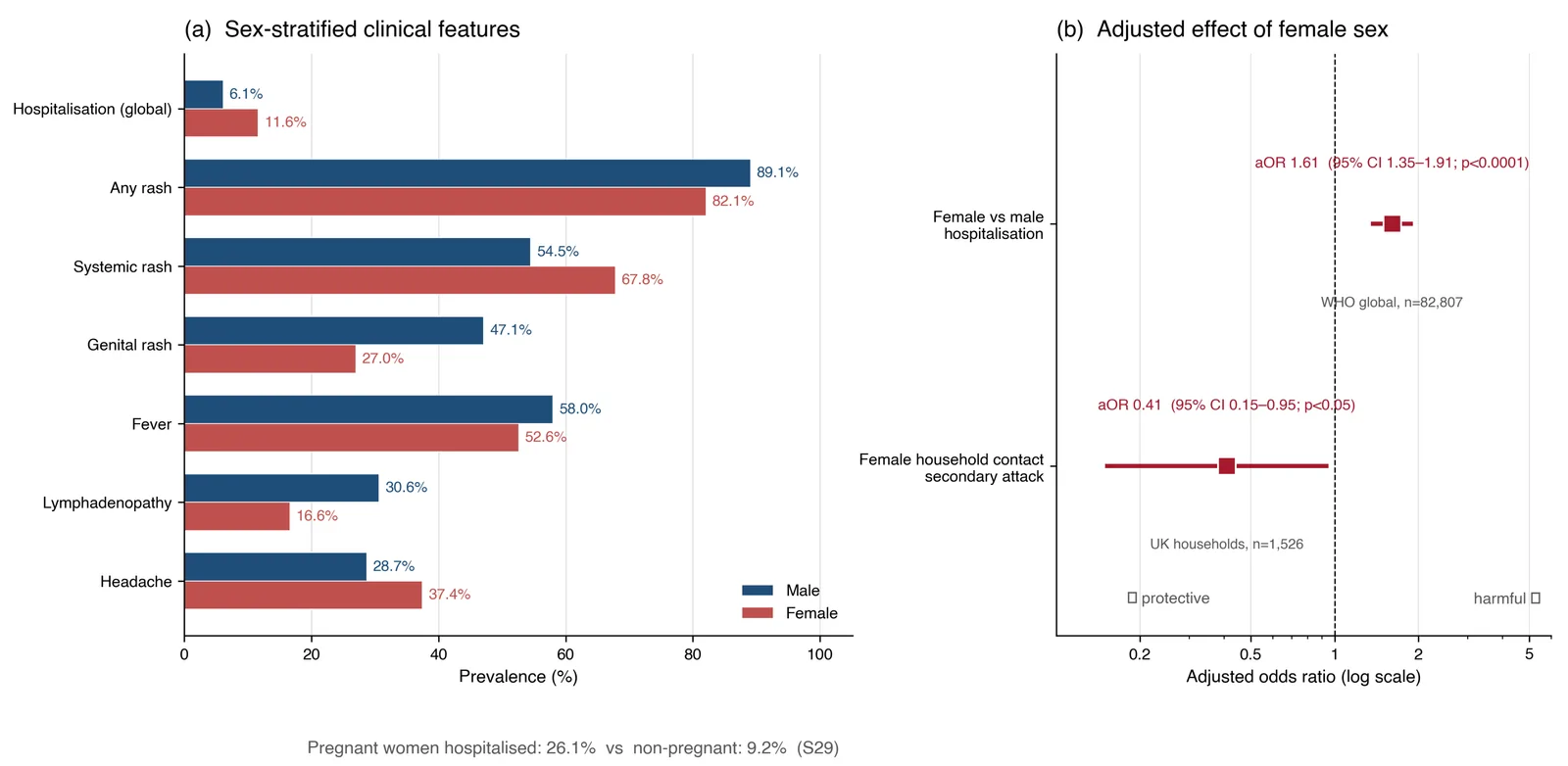
Sex and clinical outcomes in mpox. (**a**) Sex-stratified prevalence of the principal clinical features in the global WHO dataset [8] and the synthesis by Hosain et al. [3]. (**b**) Forest plot of adjusted odds ratios for the effect of female sex on hospitalisation (WHO global, n=82,807) [8] and on secondary attack rate among household contacts (UK, n=1526) [28]. The horizontal axis is log-scaled. Whiskers indicate 95% confidence intervals.

Despite their small numerical contribution, women in the European and global outbreak experienced a disproportionate burden of severe disease (Figure 6). In the WHO global analysis of 82,807 cases, the hospitalisation rate was nearly double in women (11.6%) than in men (6.1%), and after adjustment for age, HIV status and region the odds of hospitalisation in women remained 61% higher (aOR 1.61, 95% CI 1.35–1.91; p<0.0001) [8]. Pregnant women represented an especially vulnerable subgroup, with a hospitalisation proportion of 26.1% vs. 9.2% in non-pregnant women [8]. On the other hand, female household contacts in the UK study were less likely than males to develop secondary infection (aOR 0.41, 95% CI 0.15–0.95) [28], reflecting the predominantly sexual nature of household-acquired transmission during the outbreak.

Sex-specific differences extended to the clinical phenotype as well. Genital rash was nearly twice as common in men as in women (47.1% vs. 27.0%; p<0.0001), and lymphadenopathy was likewise more frequent in men (30.6% vs. 16.6%), whereas women more frequently reported systemic rash (67.8% vs. 54.5%) and headache (37.4% vs. 28.7%) [3]. These patterns are consistent with anatomically distinct primary inoculation sites in heterosexually exposed women and with a possibly later or less specific clinical presentation, both of which may contribute to the observed delay in diagnosis and the elevated hospitalisation risk.

The principal clinical implication is that although Clade IIb mpox is an MSM-predominant disease, the small minority of women infected appear to carry a higher per-case risk of severe outcomes. Clinicians in non-specialist settings must be suspicious of mpox in women presenting with compatible mucocutaneous or systemic features, and pregnancy in particular warrants close inpatient monitoring. With Clade I/Ib now being detected and locally transmitted within Europe [32,35], this risk asymmetry is expected to gain relevance, and would be amplified further if transmission broadens, because Clade I historically affects women and children at much higher proportions [4,5,31].

### 5.2. Age and Clinical Outcomes

**Figure 7:**
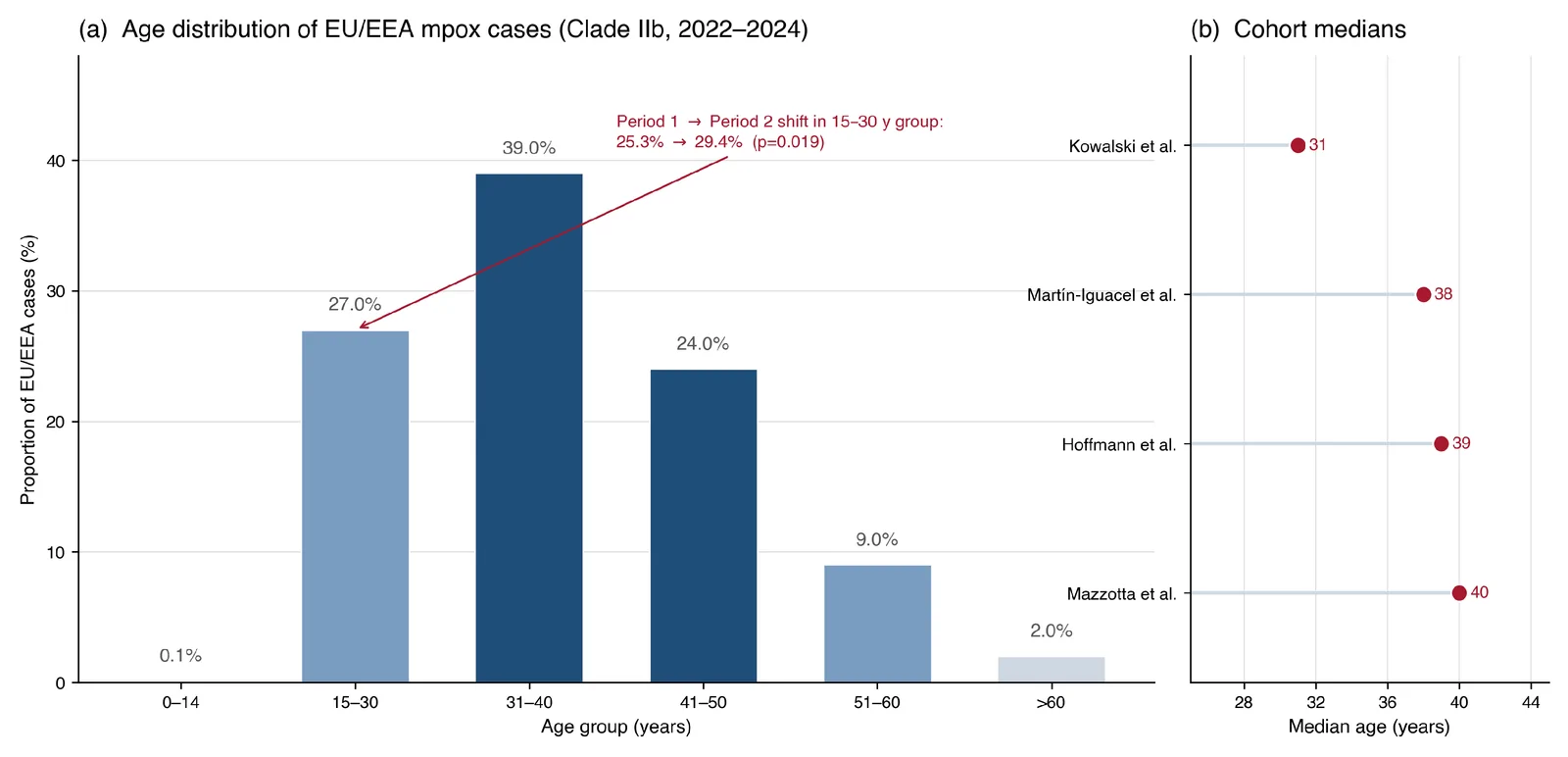
Age distribution of EU/EEA mpox cases during the Clade IIb outbreak. (**a**) Proportion of cases by age band in EU/EEA surveillance [7]. (**b**) Reported median ages in the principal national cohorts [9,10,21,26].

The age distribution of European Clade IIb cases is mainly concentrated in working-age adults (Figure 7). Across the EU/EEA, approximately 39% of cases fell within the 31–40 year band and 24% within the 41–50 year band, with paediatric cases (0–14 y) representing only 0.1% of notifications and individuals older than 60 years accounting for ∼2% [7]. Cohort median ages were remarkably consistent across countries, ranging from 31 years in the Polish hospital cohort of Kowalski and colleagues [26], through 38–39 years in the PISCIS Catalan cohort and the German multicentre cohort [9,21], to 40 years in the Italian mpox-ICONA cohort [10], reflecting the demographic profile of the sexual networks in which the outbreak propagated. Importantly, an age-structure shift was detectable between the two surveillance periods: the proportion of cases aged 15–30 rose from 25.3% in 2022 to 29.4% in 2023–2024 (p=0.019), indicating a gradual broadening or replacement of the original transmission cohort over time [7].

**Figure 8:**
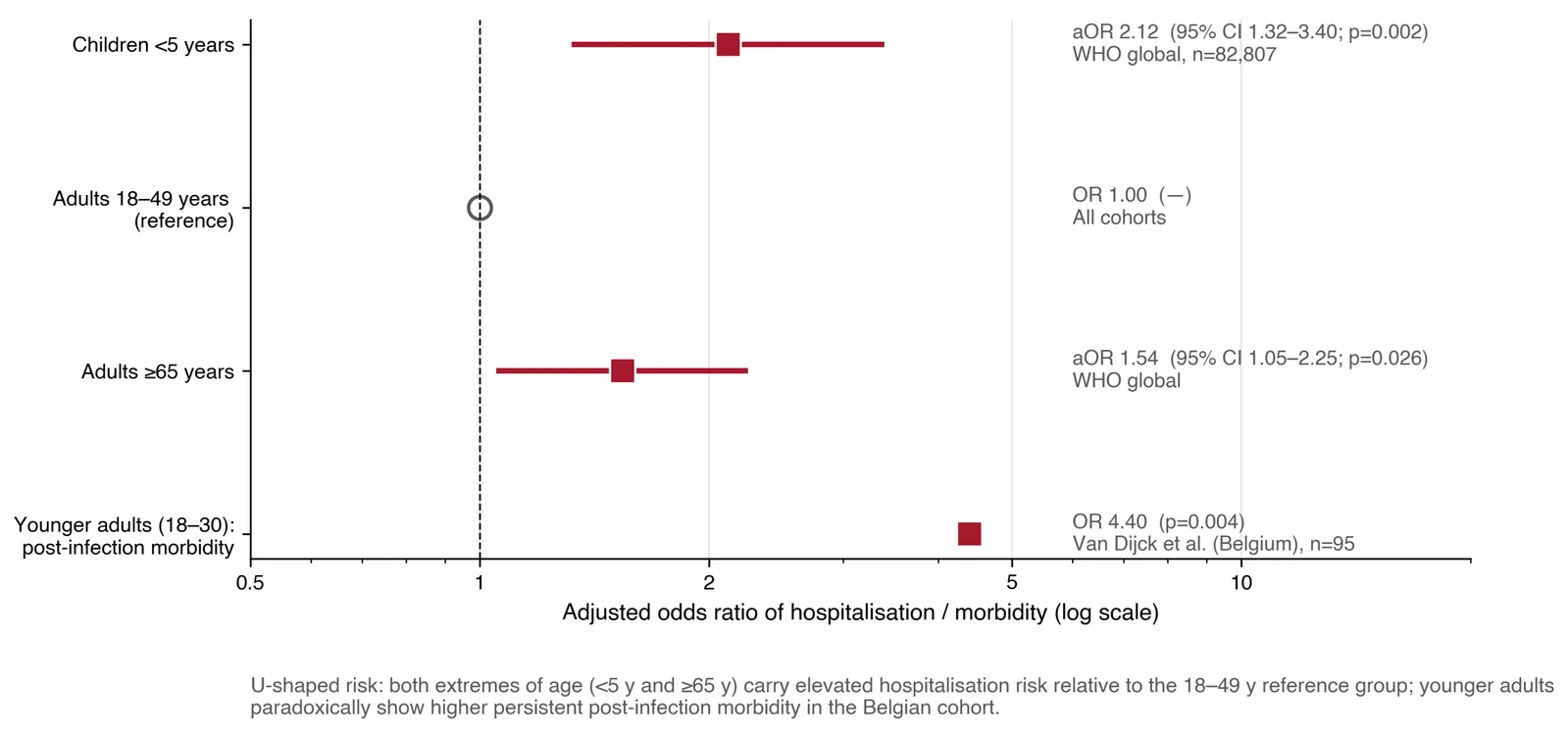
Age as a predictor of adverse mpox outcomes. Forest plot of adjusted odds ratios for hospitalisation by age group (WHO global, n=82,807) [8], together with the Belgian ITM cohort signal of 4.4-fold elevated post-infection morbidity in younger adults [24]. The horizontal axis is log-scaled. Whiskers indicate 95% confidence intervals where reported.

In contrast to the age distribution of cases, the age distribution of adverse outcomes exhibits a U-shaped pattern (Figure 8). The WHO global analysis identified two age extremes at significantly elevated risk of hospitalisation relative to the 18–49 year reference: children younger than 5 years (aOR 2.12, 95% CI 1.32–3.40; p=0.002) and adults aged 65 years or older (aOR 1.54, 95% CI 1.05–2.25; p=0.026) [8]. The clinical relevance of these point estimates in the European setting is currently limited by the very small numbers of cases at either extreme, but it has important implications for preparedness: with Clade I/Ib transmission now emerging within Europe [32,35], and set to broaden further should it become established, paediatric exposure is expected to be more frequent, given that children under 15 account for approximately 70% of cases and around 85% of fatalities in current DRC Clade Ib epidemics [4,5,31]. Partial residual immunity from historical smallpox vaccination, reported in approximately 15% of European cases aged over 40 in Period 1 versus 7% in Period 2, may attenuate severity in some older adults but provides no protection to children born after the cessation of smallpox vaccination programmes [7].

A counter-intuitive observation emerged from the Belgian ITM cohort (n=95): younger adults aged 18–30 years experienced a 4.4-fold higher risk of persistent post-infection morbidity than their older counterparts (p=0.004) [31]. This signal, although derived from a single cohort and an open question pending replication, may reflect a higher frequency of severe anorectal or genital involvement, more delayed care-seeking behaviour, or differences in baseline immune priming, and underscores that acute severity and long-term sequelae do not necessarily share the same age gradient.

To sum up, sex and age stratification of the European Clade IIb data describe a comparatively narrow at risk population in the acute phase (adult men, predominantly MSM, aged 30–50 years) but a much broader at risk distribution for severe outcomes (women, pregnant women, children < 5 years, adults ≥ 65 years) and for persistent morbidity (younger adults). Surveillance and clinical preparedness strategies should be structured accordingly, both to maintain sensitivity to ongoing Clade IIb transmission outside the predominantly MSM core and to anticipate the expected demographic shift as Clade I/Ib transmission, now emerging in Europe [32,35], broadens.

### 5.3. Geographic Location and Outcomes

Geographic variation in mpox outcomes during the 2022–2024 outbreak reveals differential case ascertainment between health system contexts, structural differences in access to HIV and sexual health services, and intra-European urban concentration of the at-risk population. EU/EEA surveillance reported a hospitalisation rate of 5.3–6.0% and a case fatality rate of 0.04% (10/22,662), whereas the WHO global synthesis recorded an apparently far higher hospitalisation proportion in the Americas (38%) at a comparable CFR [7,8] (Table 4). This may be attributed to differences in denominators rather than in severity. European cases were captured through dense sexual health service networks, inflating the mild-disease denominator, whereas hospital-based reporting predominated elsewhere. Hospitalisation data in mpox must be interpreted strictly within their ascertainment context, and cross-regional rate comparisons must be made with caution. HIV co-infection in DRC mpox cases is bimodal, kept low in the paediatric Clade Ia case but substantial (25–30%) in the adult Clade Ib sexual transmission cluster identified in South Kivu in 2023–2024, where transmission is concentrated in sex workers and their contacts.

Within Europe, transmission was mainly concentrated in major urban centres (i.e., London, Madrid, Barcelona, Paris, Amsterdam, Berlin and Milan), a pattern validated in the UK programme, in which 71% of MVA-BN doses were administered in London alone [28]. Spain and Portugal carried the highest early burden, with Spain remaining the leading EU country across both surveillance periods, while Germany, France, Italy and the UK sustained continuous low-level transmission [7]. Within-country heterogeneity was equally important. The Spanish primary care detection programme of Martínez-Arias et al. [18] identified a clinically distinct case population (younger, more female, more heterosexual) that was systematically under-ascertained by specialist sexual health clinics, suggesting that the European case profile derived from hospital-based surveillance is not fully representative. A further intra-cohort gradient was observed in the PISCIS Catalan dataset, in which 21% of cases were of Latin American origin and all 12 people with HIV (PWH) with CD4 < 200 cells/μL belonged to this group (p<0.0001) [21]. The same cohort found an order of magnitude difference in hospitalisation between CD4 < 200 and CD4 ≥ 200 cases (16.7% vs. 2.0%), placing geographic inequities in access to durable HIV care as the dominant driver of severe mpox outcomes within Europe.

### 5.4. Ethnicity and Socioeconomic Factors

Ethnicity and socioeconomic predictors of mpox outcomes are under-reported in the European literature, and most cohorts report only descriptive ethnic composition without outcome stratification. However, two signals emerge and merit prospective evaluation. First, the ICONA Italian multicentre cohort and the Catalan population-wide study both reported higher risk of severe disease among Caucasian patients (OR ≈ 1.82) [10,21]. This counter-intuitive direction of effect is unlikely to reflect a true biological gradient and is probably attributable to differential healthcare-seeking behaviour, differential access to specialist sexual health services, or to confounding by undetected, uncontrolled HIV in non-Caucasian groups. In the PISCIS Catalan cohort, the entirety of the CD4 < 200 PWH stratum, the population at by far the highest risk of severe and disseminated mpox was of Latin American origin [21], indicating that structural barriers to HIV diagnosis, linkage to care and anti-retroviral therapy in migrant populations translate directly into the most severe mpox phenotypes observed in Europe. The WHO global analysis did not report ethnicity-stratified European outcomes [8], and no reviewed European cohort has performed a multivariable analysis incorporating ethnicity, country of birth, language barriers, insurance status or income. In such a case, the relationship between migration, equitable access to HIV care, and mpox severity represents a critical, evidence-poor dimension of health equity that should be a priority for future prospective study design.

### 5.5. Multivariable Predictors of Severity: Synthesised Evidence

Synthesising the adjusted analyses reported across the reviewed European and global cohorts produces a coherent hierarchy of predictors for severe mpox, as seen in Table 5. Effect sizes are adjusted odds ratios (aORs) or hazard ratios (HRs) when reported. The dominant signal is immunological, where HIV-negative iatrogenic or disease-related immunosuppression carries the largest effect size of any single predictor (aOR 3.47, 95% CI 1.84–6.54), followed by uncontrolled HIV infection (aOR 2.00, 95% CI 1.68–2.37) [8]. Within the PWH stratum, CD4 count < 200 cells/μL stands out as the clearest single severity marker available in routine European practice, associated with an 8-fold higher hospitalisation rate and nearly doubled prevalence of generalised rash relative to CD4 ≥ 200 in the PISCIS cohort [21]. Conversely, in the multinational MOSAIC cohort, in which most participants with HIV had preserved CD4 counts and were receiving antiretroviral therapy, HIV status did not materially alter clinical presentation [13], reinforcing that it is advanced or uncontrolled HIV that drives severe disease. Demographic predictors (i.e., very young age (<5 y), older age (≥65 y), female sex and pregnancy) have smaller risk increments (Section 5.1 and Section 5.2). Clinical presentation features are usefully predictive at first contact. For instance, in the ICONA cohort, presenting fever and lymphadenopathy were each independently associated with approximately a 2-fold increase in the odds of a severe course [10]. Finally, prior sexually transmitted infection in the preceding 12 months carried the highest risk for incident mpox in the Danish MSM-HIV cohort (HR 7.1, 95% CI 1.9–26.9), identifying recent STI as a practical, actionable target for pre-exposure vaccination prioritisation in MSM/HIV populations [25].

Three practical implications follow directly from this synthesis. First, immune status, whether measured as HIV viral suppression, CD4 count, or non-HIV immunosuppression, is the most informative parameter for severity stratification at first clinical contact and must drive both inpatient triage and antiviral therapy decisions. Second, demographic predictors at the extremes of age and in pregnant women confer real but smaller risk increments, and become quantitatively more important as the case-mix broadens beyond the predominantly adult MSM Clade IIb outbreak demographic, particularly as Clade I/Ib transmission becomes established in Europe. Third, prior STI history provides a readily ascertainable, evidence-based criterion for vaccination prioritisation in the MSM/HIV population and is, to our knowledge, the strongest single behavioural/clinical predictor of incident mpox reported in any European cohort. Together, these predictors define an evidence-grounded, deployable severity framework that can be operationalised in routine European sexual health and infectious disease practice.

## 6. Antiviral Therapy: Safety and Efficacy

Antiviral therapy for mpox during the 2022–2024 European outbreak was mainly characterised, in both clinical practice and the regulatory conversation, by tecovirimat, the only oral small-molecule developed to target an orthopoxvirus protein. Four agents have been proposed in clinical mpox practice (Table 6). Tecovirimat (ST-246) inhibits the orthopoxvirus VP37 protein and blocks production of extracellular enveloped virions, suppressing cell-to-cell spread. It remains the only agent in the EU with a regulatory indication that explicitly encompasses orthopoxvirus disease [1,38]. Cidofovir and its oral prodrug, brincidofovir, act earlier in the replication cycle by inhibiting viral DNA polymerase and have been used under compassionate use provisions despite the absence of mpox-specific licensure [1,2,3]. Vaccinia immune globulin (VIGIV) provides passive antibody and is reserved for vaccine complications and the most severe presentations. However, the EMA restricted the approved use of tecovirimat for mpox treatment in March 2026 following the accumulation of negative randomised trial data and concerns about the emergence of resistance [45]. The agent remains available through restricted programs for selected severe or immunocompromised cases.

### 6.1. Tecovirimat

Preclinical evidence for tecovirimat is consistent and robust. In vitro and lethal orthopoxvirus animal models, including non-human primate studies, reveal suppression of viral replication and reduced mortality, and their combination with ACAM2000 vaccination has shown additive protection [1,2,3]. This animal-model evidence underpinned both the original FDA smallpox licensure and the compassionate-use frameworks under which the agent entered clinical mpox practice.

**Figure 9:**
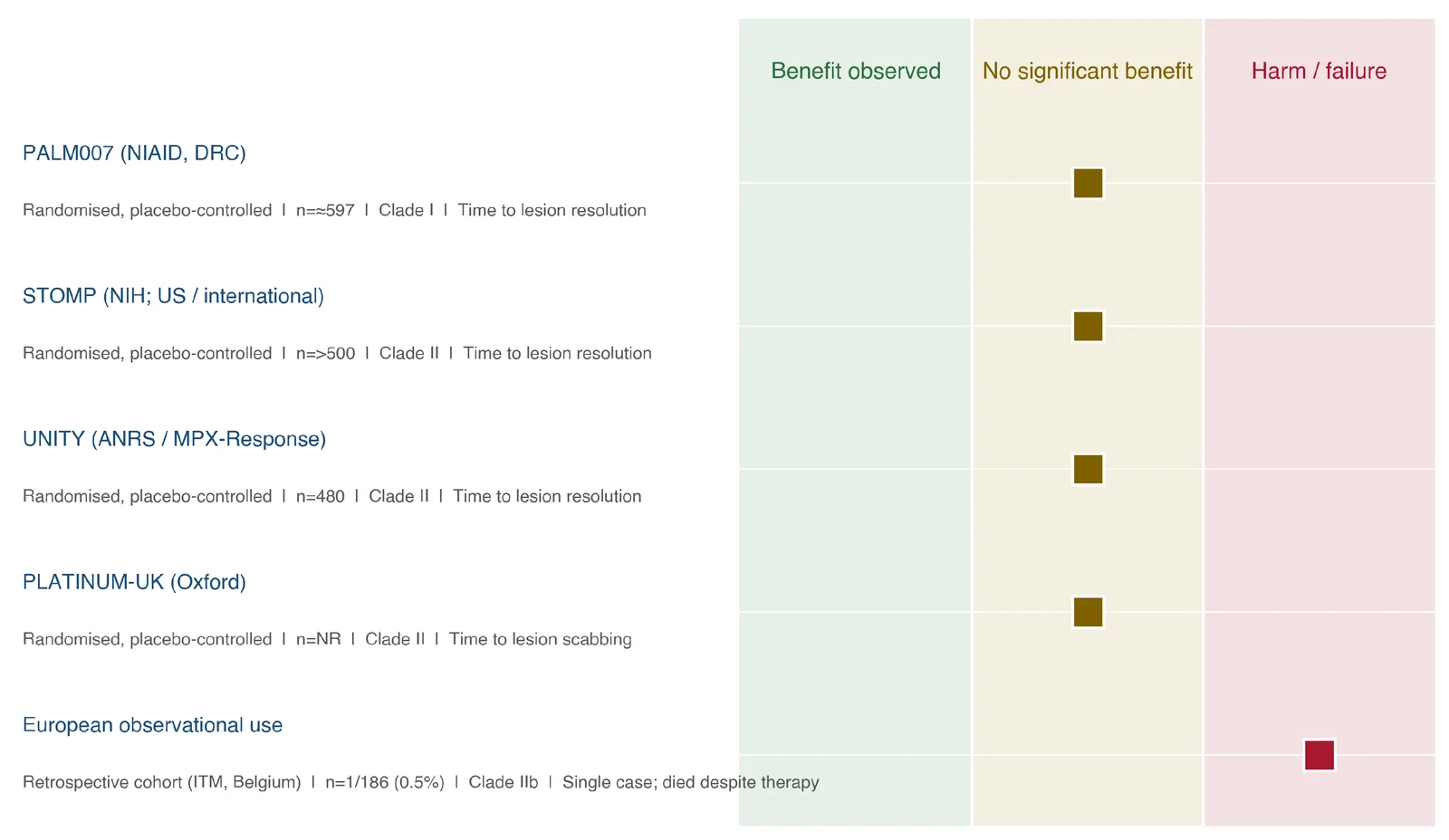
Qualitative summary of pivotal trial outcomes for tecovirimat in mpox. Each row summarises one pivotal randomised placebo-controlled trial (PALM007, STOMP, UNITY, PLATINUM-UK) or the European observational record (ITM, Belgium). The marker is placed in the categorical zone corresponding to that study’s reported outcome: “benefit observed”, “no significant benefit”, or “harm/failure”. Marker position is categorical only and does not encode a quantitative effect size or confidence interval.

The pivotal randomised clinical evidence, however, is uniformly negative (Figure 9). Four randomised, double-blind, placebo-controlled trials have now reported, one in Clade I disease and three in Clade II. PALM007 (NIAID, DRC) randomised 597 patients with PCR-confirmed Clade I mpox to tecovirimat or placebo and found no reduction in time to lesion resolution and no mortality benefit [39]. In Clade II disease, the NIH-sponsored STOMP trial in a predominantly immunocompetent international population [40], the ANRS/MPX-Response UNITY trial (480 participants in Argentina, Brazil and Switzerland) [41], and the UK PLATINUM-UK trial [42] each likewise failed to show any benefit over placebo on time to lesion resolution (or, for PLATINUM-UK, time to lesion scabbing), with virological and pain secondary endpoints also null. Across all four trials, tecovirimat was safe and well tolerated, with an adverse-event profile comparable to placebo [39,40,41]. European observational use was, by contrast, extremely sparse: only a single patient in the 186-case Belgian ITM cohort received tecovirimat, and that patient died despite therapy [21,24]. This convergent negative evidence across four randomised trials formed the explicit basis for the regulatory action that followed.

In March 2026, the European Medicines Agency’s Committee for Medicinal Products for Human Use (CHMP), acting on a referral initiated by the European Commission, recommended that tecovirimat SIGA should no longer be used for the treatment of mpox, having reviewed all available data from the four randomised trials above (PALM007, STOMP, UNITY and PLATINUM-UK) [45]. The recommendation on other authorised orthopoxvirus indications (smallpox, cowpox, complications of smallpox vaccination), remain unaffected [45].

**Figure 10:**
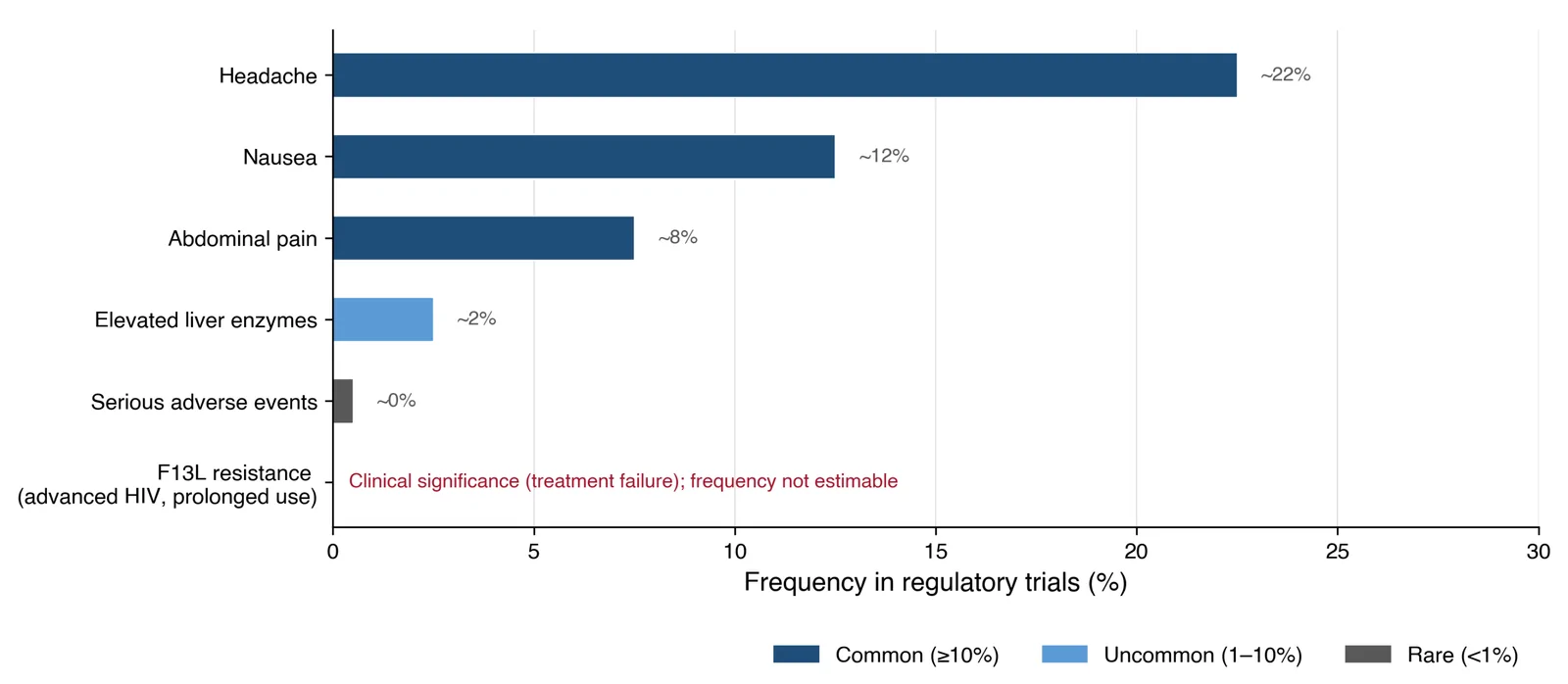
Tecovirimat safety profile in regulatory trials. Common, typically mild gastrointestinal and neurological adverse events dominate the standard tolerability profile; the most consequential signal is the emergence of F13L resistance in patients with advanced HIV receiving prolonged therapy [43,44].

Tecovirimat is generally well tolerated in immunocompetent adults enrolled in regulatory trials (Figure 10). The commonest treatment-emergent adverse events are mild and self-limiting, such as headache (∼20–25%), nausea (∼10–15%) and abdominal pain (∼5–10%), with elevated liver enzymes occurring uncommonly and serious adverse events rare [38]. The clinically dominant safety signal, however, is not an idiosyncratic toxicity but the emergence of resistance. Case reports in patients with advanced HIV receiving prolonged tecovirimat monotherapy have documented mutations in the VP37-encoding F13L gene that abolish drug activity and lead to treatment failure [43,44]. Because the highest-need patients are those most likely to receive prolonged courses, the resistance signal materially constrains the appropriate clinical use of the agent.

**Figure 11:**
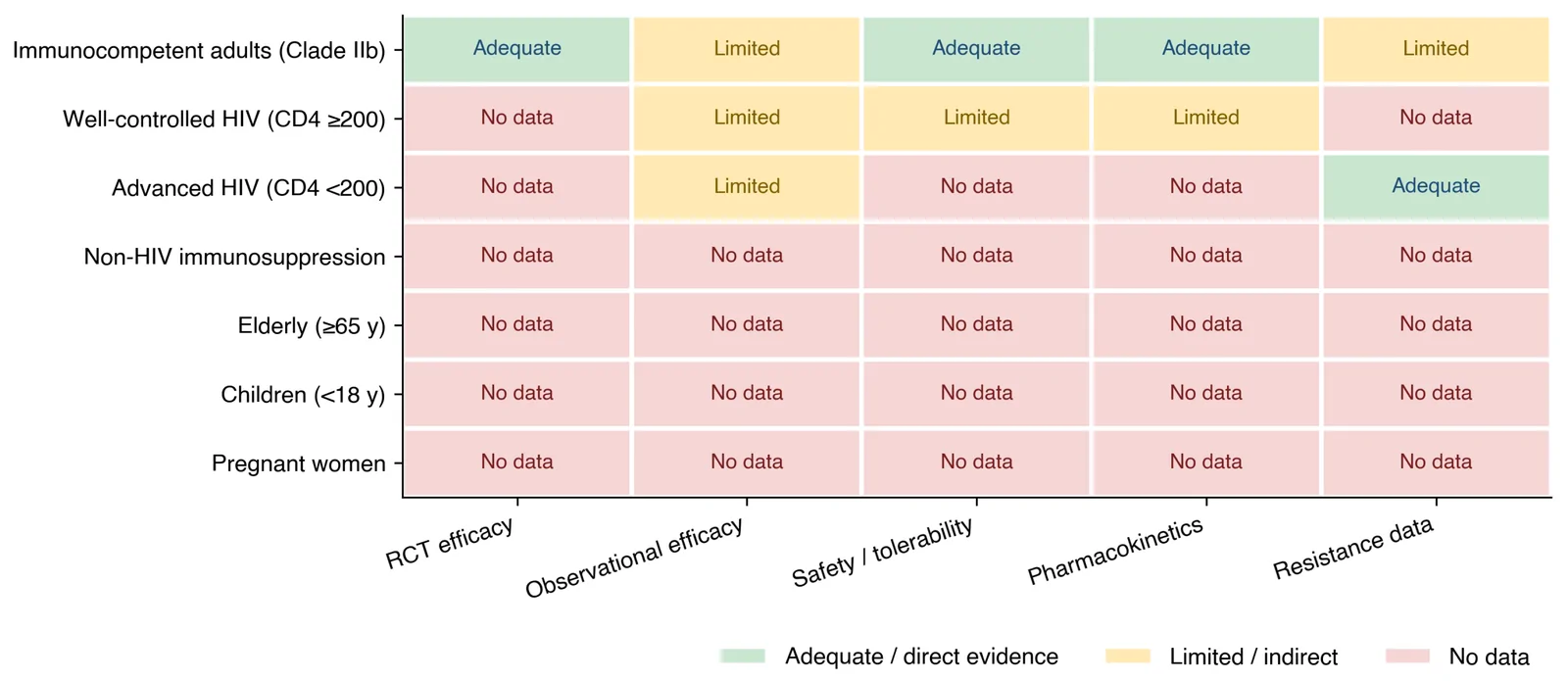
Tecovirimat: evidence-availability heatmap across at-risk subgroups. Cells encode the maturity of the reviewed evidence base, from direct adequate evidence (green) through limited or indirect (amber) to no data (red). The patient groups at greatest risk of severe mpox, non-HIV immunosuppression, the elderly, children and pregnant women, are precisely those for which the antiviral evidence base is least developed.

The most striking feature of the tecovirimat evidence base is its asymmetric distribution across the patient subgroups in which the agent would be most clinically indicated (Figure 11). For immunocompetent adults with Clade IIb disease, the evidence is adequate, although ultimately negative. For people with well-controlled HIV, the evidence is limited and indirect; for people with advanced HIV (CD4 < 200 cells/μL), the only direct evidence is the resistance signal itself [43,44]. For non-HIV immunosuppression, the subgroup with the highest reported adjusted odds of severe mpox (aOR 3.47) [8], no efficacy or safety data exist in any reviewed paper. The same is true of elderly patients (≥65 y), children, and pregnant women, despite the fact that paediatric patients account for an estimated 70% of cases and around 85% of deaths in the ongoing African Clade Ib epidemic [4,5,31]. With Clade I/Ib now being detected and locally transmitted within the EU/EEA [32,35], these untested subgroups are no longer a hypothetical African concern but a foreseeable European one.

The clinical implication is uncomfortable but consistent across the evidence: the European Clade IIb outbreak generated substantial clinical experience of mild, self-resolving disease in which tecovirimat was neither indicated nor necessary, while the subgroups in whom the agent might plausibly confer benefit remain effectively untested. The resistance literature further argues that tecovirimat monotherapy in patients with profound and persistent immunosuppression is unlikely to be a durable strategy. Closing this evidence gap, ideally through adaptive trial designs prioritising advanced HIV, paediatric Clade I exposure and pregnancy, is the single most important research priority arising from this review.

### 6.2. Cidofovir and Brincidofovir

**Figure 12:**
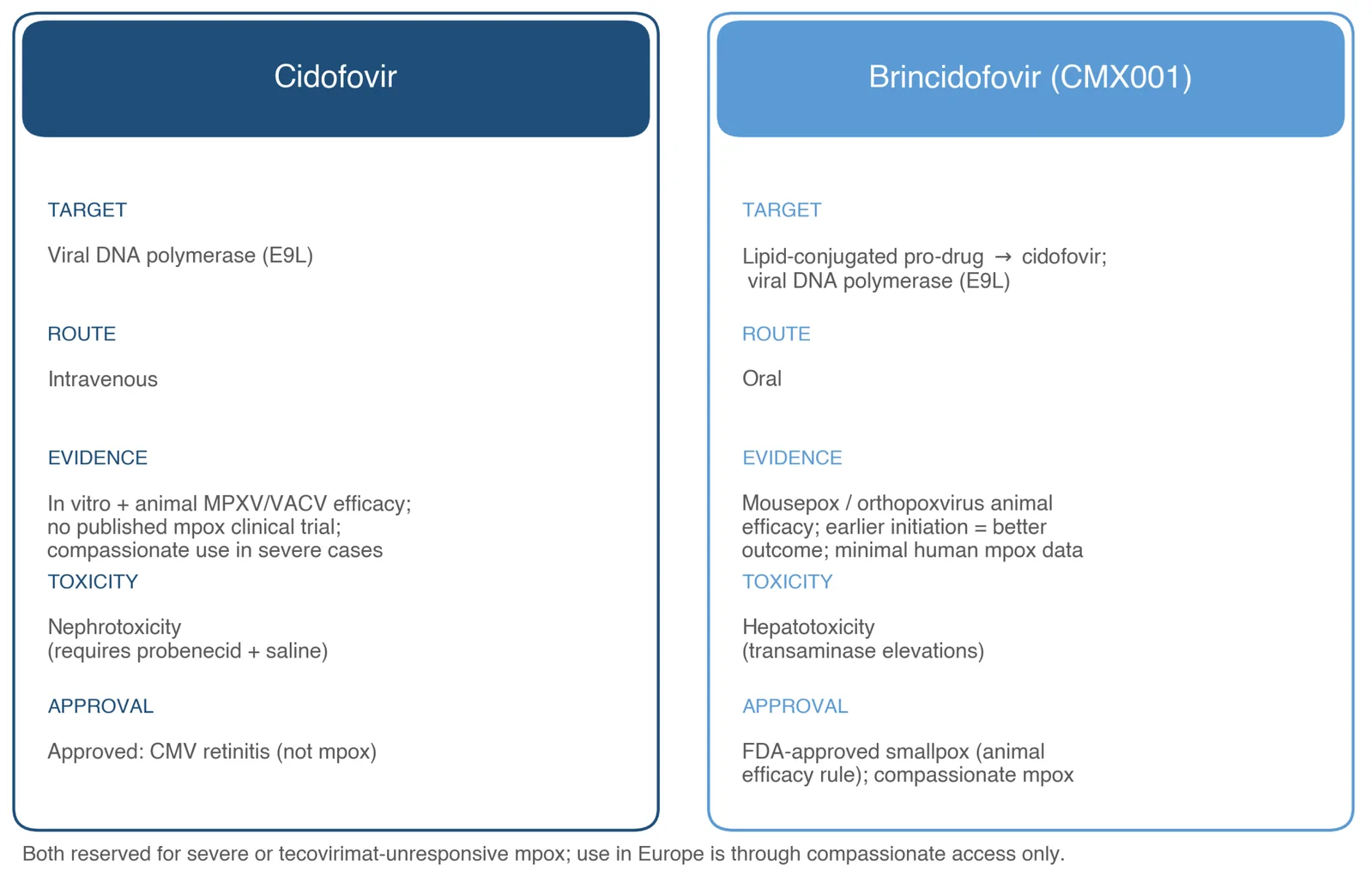
Comparative profile of cidofovir and brincidofovir for mpox. Both agents inhibit viral DNA polymerase (E9L), providing a mechanism distinct from that of tecovirimat, but human mpox-specific clinical data remain limited, and organ-specific toxicity is substantial.

Cidofovir and its oral lipid-conjugated pro-drug brincidofovir are the principal second-line orthopoxvirus antivirals available in Europe. Both target the viral DNA polymerase (encoded by the E9L gene), acting at a step of the replication cycle distinct from tecovirimat, making them mechanistically attractive as candidates for combination or salvage therapy. Figure 12 summarises their profiles. The clinical evidence base for both agents in mpox, however, is distinctly thin. Efficacy data derive primarily from in vitro experiments and animal models of mousepox and vaccinia, and no randomised trial has demonstrated benefit in human mpox [1,2,3]. The two agents also carry non-trivial organ-specific toxicities, nephrotoxicity for intravenous cidofovir, hepatotoxicity for oral brincidofovir, that further restrict their routine use. Within Europe, both are accessible only through compassionate-use channels and are reserved for severe or tecovirimat-unresponsive presentations, particularly in immunocompromised patients, where the benefit-to-risk balance may justify their use [3,38].

### 6.3. Vaccinia Immune Globulin Intravenous (VIGIV)

VIGIV delivers passive orthopoxvirus antibody and is approved by regulatory authorities for severe complications of vaccinia vaccination. Compassionate use in severe mpox has been reported anecdotally, and the agent is occasionally considered as adjunctive therapy in profoundly immunocompromised patients failing small-molecule antivirals, but no structured efficacy or safety data for mpox exist in any reviewed paper, and no mpox-specific dosing recommendation has been validated [3,38]. Its place in the European treatment algorithm, therefore, remains empirical and salvage-oriented.

### 6.4. Antiviral Resistance

**Figure 13:**
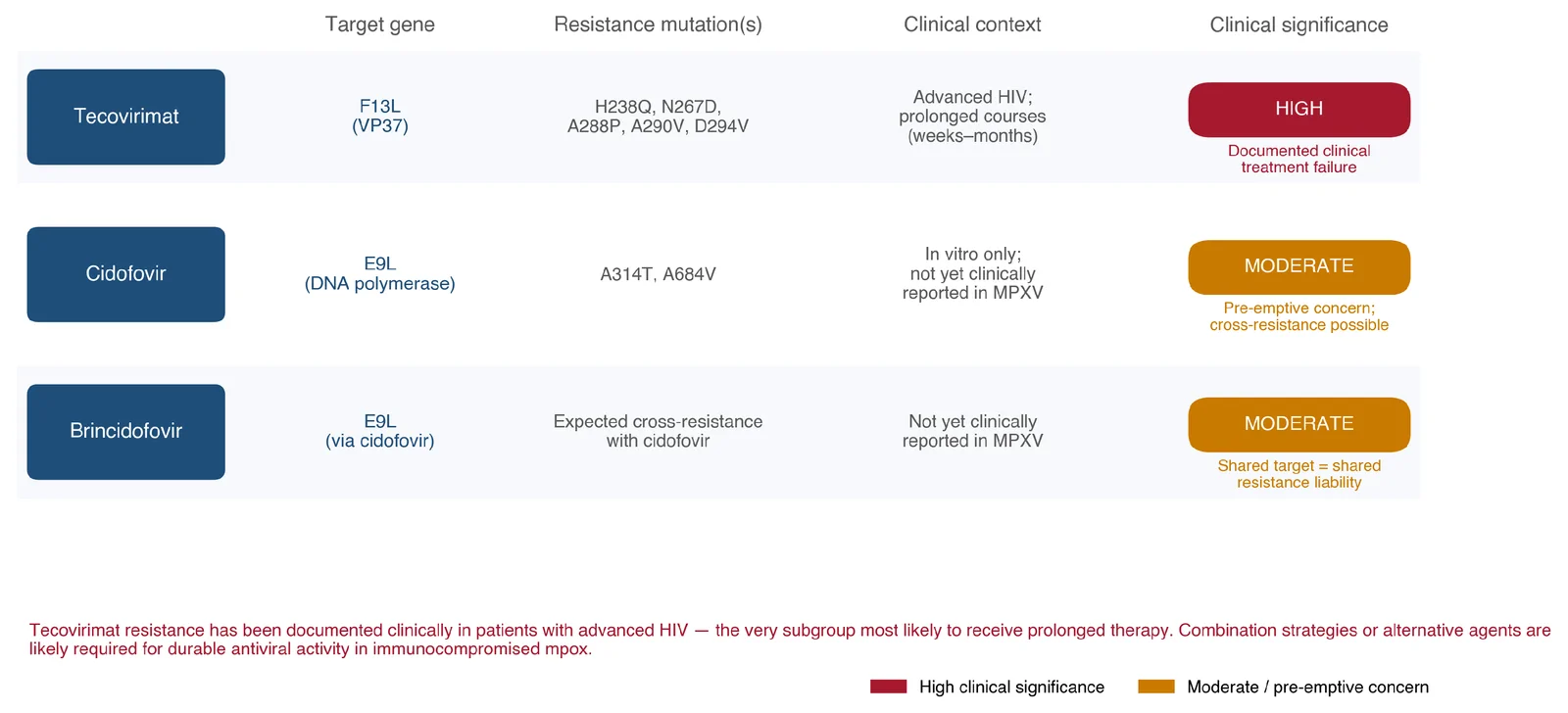
Antiviral resistance landscape in mpox. Tecovirimat resistance via F13L mutation is the only clinically documented resistance event to date and has occurred in patients with advanced HIV on prolonged therapy [43,44]. Cidofovir and brincidofovir share the E9L target and therefore an anticipated cross-resistance liability, although clinical MPXV resistance has not been reported.

The main output across the reviewed antiviral evidence base is the emergence of clinically relevant resistance to tecovirimat (Figure 13). Single amino-acid substitutions in the F13L gene, which encodes the VP37 envelope protein targeted by tecovirimat, abolish drug activity and have been documented in patients with advanced HIV receiving prolonged therapy [43,44]. For cidofovir and brincidofovir, the resistance signal is, for now, pre-emptive rather than clinical: mutations in the E9L DNA polymerase gene (notably A314T and A684V) reduce drug susceptibility in vitro, and their shared target predicts cross-resistance, but neither has yet been clinically reported in MPXV [60].

There is one clinical paradox: the patients most likely to require prolonged antiviral therapy, those with profound and persistent immunosuppression, are precisely those in whom tecovirimat monotherapy has been shown to fail through resistance. Combined with the negative pivotal randomised data from Section 6.1, this argues against tecovirimat monotherapy as a durable strategy in this population. Practical implications include earlier consideration of mechanistically non-overlapping agents (cidofovir or brincidofovir) as combination or salvage therapy in selected severe cases, the need for systematic genotypic surveillance of F13L in patients receiving extended courses, and the prioritisation of adaptive trials of combination antiviral regimens in immunocompromised mpox cohorts as the most actionable research gap arising from this study.

## 7. Evidence Gaps and Research Priorities

**Figure 14:**
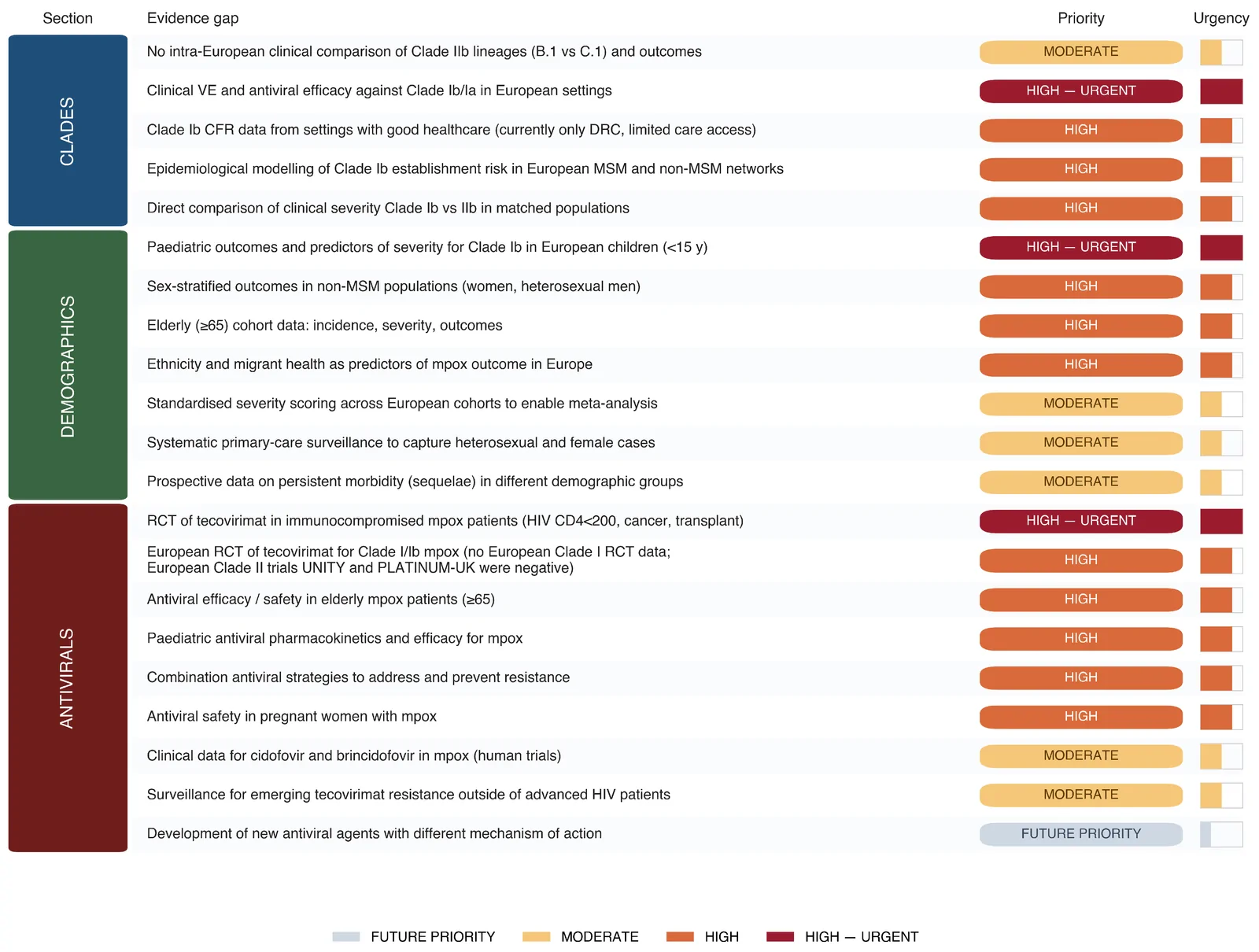
Research priorities across the three review axes (clades, demographics, antivirals). Each row is one of the 21 evidence gaps identified in the reviewed European literature. Colour denotes a consensus priority tier, from hlfuture priority to high/urgent, based on each gap’s bearing on severe outcomes and the scarcity of European data, not on a measured value.

From the reviewed literature, we identified 21 specific evidence gaps and grouped them into the review’s three axes, five for viral clades, seven for demographics, and nine for antiviral therapy (Figure 14). These questions do not share a common quantitative metric; we only rank each gap by two simple criteria, i.e., how directly it bears on severe outcomes and how little European evidence currently exists, yielding four ordinal tiers (future priority, moderate, high, and high/urgent). The output is a structured summary of that judgement.

Two priorities stand out. First, the clinical and demographic profile of Clade I/Ib in Europe is essentially unknown. Its severity and paediatric burden have been characterised almost entirely through surveillance in the DRC, where access to care differs greatly from that in European settings, and no European data yet allow a direct comparison between Clade Ib and Clade IIb. This gap is now pressing as Clade I/Ib is being detected in Europe, apart from imported cases [4,5,8,31,33]. Moreover, antiviral evidence is asymmetric, meaning that the largest body of data describes the mild, self-limiting Clade IIb disease in which antivirals are seldom clinically necessary, while the subgroups in whom antivirals would plausibly be most beneficial (i.e., patients with advanced HIV, non-HIV immunosuppression, children, pregnant women and the elderly), remain effectively untested by randomised data [8,21,43,44,53].

A focused research agenda follows from these gaps: (i) adaptive, multi-arm randomised trials of tecovirimat, and of combinations with cidofovir or brincidofovir, that prioritise immunocompromised, paediatric and pregnant patients, (ii) a standardised European severity score so cohorts can be pooled and compared, and (iii) real-time genomic surveillance for both Clade I/Ib spread and tecovirimat F13L resistance. Closing the highest-priority gaps in Figure 14 would address the greatest weakness in current European mpox care and should be a near-term goal for academic networks and public health funders.

## 8. Conclusions

The primary finding of this review is a concerning mismatch in the current evidence. Most European research focuses on mild cases in otherwise healthy individuals. However, there is a substantial evidence gap for the exact populations that require the most medical intervention, such as patients with advanced HIV, non-HIV immunosuppression, pregnant women, children, and the elderly.

The 2022–2024 mpox outbreak in Europe was driven solely by Clade IIb, which exhibited a high survival profile and a very low case-fatality rate of 0.04%. The progression from the first imported Clade Ib case in Sweden in August 2024 to confirmed Clade I detections across multiple EU/EEA countries and documented secondary local transmission in the UK by 2026 underscores that geographical expansion is now underway. For travel medicine and infectious disease practice, the relevant question is no longer whether Clade I/Ib MPXV can be imported into Europe, but how reliably clinicians and public-health systems can identify cases after travel-related introduction, detect autochthonous spread, protect higher-risk groups, and use a narrowing antiviral armamentarium responsibly.

Within the European context, the epidemic has been mainly concentrated among adult MSM in urban networks. However, the critical determinant of severe morbidity is immunological, not only strictly demographic. Individuals with uncontrolled HIV and a CD4 count below 200 cells/μL face significantly disproportionate hospitalisation rates. Furthermore, this biological vulnerability is severely compounded by structural health inequalities, particularly among migrant populations. As the global epidemiological landscape shifts with the spread of Clade Ib, clinical vigilance must be expanded to encompass populations currently under-represented in European data but recognised globally as carrying significantly elevated risks, including children under five, older adults, women, and pregnant individuals.

Finally, the current therapeutic armamentarium for mpox remains inadequate. The failure of tecovirimat to demonstrate clinical effectiveness in robust randomised trials across both Clade I and Clade IIb settings, culminating in its restriction by the EMA in March 2026, highlights a critical gap in clinical management. Compounded by the emergence of tecovirimat resistance in patients with advanced HIV, and an absence of clinical trial data for alternatives like cidofovir or brincidofovir, the most vulnerable subgroups are left with the least evidence-based options. Addressing this therapeutic gap and prioritising inclusive, rigorous clinical research for severely immunocompromised patients and high-risk demographics must be the central priority for global mpox preparedness.

## Figures and Tables

**Table 1:** Summary of recent reviews, meta-analyses, and guidelines highlighting European mpox outcomes and preparedness (2025–2026).

Ref.	Study	Focus Area	Key European Findings
[50]	Clinical guidelines	EACS guidelines, HIV/mpox co-infection	Version 13.0 revises mpox co-infection protocols and incorporates recent European clinical trial data on tecovirimat use.
[51]	Systematic review & Meta-analysis	Global and regional CFR rates	Europe: lowest regional mpox CFR rate globally (0.1%), Africa: 6.3%.
[52]	Systematic review & Meta-analysis	Hospitalization rates and clinical reasoning	Europe has the lowest pooled hospitalisation rate globally. Severe pain and bacterial superinfection drove admissions.
[53]	Scoping Review	European cohort evaluation	Found 49 cohorts. Highlighted severe data heterogeneity preventing meta-analyses.
[54]	Systematic review of reviews	Healthcare facility outbreak response	Identified systemic gaps in European healthcare facilities (UK, Italy, Spain, France), i.e., space repurposing, task-shifting, and workflow strain.
[55]	Comprehensive (narrative) review	Global expansion and viral evolution (2022–2024)	Places the European Clade IIb wave within the wider 2022–2024 evolutionary trajectory and stresses continued evolutionary and re-emergence potential.
[56]	Case report	Mpox in PrEP users (Croatia)	Presents ongoing European case detection in sexual health/PrEP populations. Notes the need for sustained clinical vigilance.
[57]	Consortium report	Genomic surveillance (Project ODIN)	Proposes a Euro/African consortium to advance near real-time environmental genomic surveillance for mpox.

**Table 2:** Epidemiological notes by country for the European mpox outbreak (predominantly Clade IIb, emerging Clade I detections noted). Cohort listing covers EU/EEA countries with published Clade IIb cohorts; an additional seven EU/EEA countries (Austria, Czechia, Greece, Ireland, Luxembourg, Norway, Romania) reported Clade I cases without an indexed cohort series in the review window [33].

Country	Epidemiological Notes and Cohort Specifics	Refs.
Spain	Most affected EU country, ongoing low-level circulation despite outbreak suppression.	[7,18,19,20,21,22]
Portugal	Early outbreak epicentre, rapidly achieved control after the initial peak.	[7,8]
Germany	Maintained the second largest EU burden with evidence of continuing transmission.	[7,9]
France	Transmission concentrated mainly around the Paris metropolitan area.	[7,12]
Netherlands	Significant burden with well-described clusters centralised in Amsterdam.	[7,8]
UK	London-centric Clade IIb transmission network (71% of national vaccine doses administered in London), 48 confirmed Clade Ib cases to April 2026, including secondary local transmission.	[7,28,29,30,35]
Italy	Tracked via multicentre prospective studies: ICONA (n=541), Moschese (n=32), Nozza (n=162).	[10,14,15,16,17,58,59]
Poland	Atypical presentation of the C.1 lineage documented in 2025 (Zmora case report).	[26,27]
Belgium	Well-studied population via multiple prospective cohorts (ITM/mpox cohort studies).	[23,24]
Denmark	High incidence (1.8%) within the Svartstein MSM/HIV longitudinal cohort (n=727).	[25]
Sweden	First EU/EEA Clade Ib case, August 2024.	[4,32]

**Table 3:** Comparative clinical and epidemiological parameters across MPXV clades.

Parameter	Clade IIb (European Outbreak)	Clade Ib (Africa 2024; Europe 2024–2026)	Clade Ia (Endemic DRC)	Refs.
Case fatality rate (CFR)	0.09–0.19%	∼3–11%, improved to 1.4–1.7% with supportive care	3–10% historically	[1,2,3,4,5,6,7,8,31]
European detections (*n*)	>22,000 confirmed (EU/EEA, 2022–2024)	608 across 18 EU/EEA countries (June 2025–May 2026); 48 in the UK up to 30 April 2026	Imported only, no established local transmission	[33,35]
EU/EEA deaths (*n*)	10 deaths in 22,662 cases 2022–2024(0.04%), 1 more Clade II death June 2025–May 2026.	0 reported to date	0 in EU	[4,7,31,33,35]
Paediatric burden	<1% of EU cases	70% of DRC cases in children <15 y	Mainly children (historical)	[4,5,7,8,31]
Fatalities in children	<1%	85% (DRC 2024)	High	[3,4,5,31]
Severity of skin disease	Predominantly limited, self-resolving	More severe in some reports	More severe	[3,8,10,13,14,27]
Transmission route	Sexual (94–96%), primarily MSM	Sexual/non-sexual, wider demographics	Mainly zoonotic and household	[1,6,7,8,9]

**Table 4:** Comparative hospitalisation, CFR and HIV co-infection across geographic regions during the 2022–2024 mpox outbreak.

Region	Hospitalisation	CFR	HIV Prevalence	Refs.
EU/EEA	5.3–6.0% (0.1% ICU)	0.04% (10/22,662)	37.8%	[7]
Americas (global data)	38.0% (reported)	variable	52.4%	[8]
Global overall	4.0%	0.1% (89/82,807)	48.0%	[8]
DRC/Central Africa (Clade I)	limited data	3–11%	25–30% (adult Clade Ib), low in paediatric Clade Ia	[3,4,5,31]

**Table 5:** Synthesised multivariable predictors of severe mpox derived from European cohort and global surveillance data.

Predictor	Effect Size	Population	Refs.
HIV-negative immunosuppression	aOR 3.47 (95% CI 1.84–6.54)	Global (n=82,807)	[8]
Uncontrolled HIV (vs HIV-negative)	aOR 2.00 (95% CI 1.68–2.37)	Global	[8]
CD4 < 200 vs. ≥200	Hospitalisation 16.7% vs. 2.0% (p=0.001), generalised rash 83.3% vs. 44.6% (p=0.008)	Catalonia (n=673)	[21]
Age < 5 years	aOR 2.12 (95% CI 1.32–3.40)	Global	[8]
Age ≥ 65 years	aOR 1.54 (95% CI 1.05–2.25)	Global	[8]
Female sex	aOR 1.61 (95% CI 1.35–1.91)	Global	[8]
Pregnancy	Hospitalisation 26.1% vs. 9.2%	Global	[8]
Fever at presentation	OR 1.95 (95% CI 1.27–2.99)	Italy (n=541)	[10]
Lymphadenopathy at presentation	OR 2.30 (95% CI 1.52–3.48)	Italy (n=541)	[10]
Caucasian ethnicity (counter-intuitive)	OR ∼1.82	Italy Belgium	[10,21]
Prior STD in the last 12 months	HR 7.1 (95% CI 1.9–26.9)	Denmark MSM/HIV (n=727)	[25]

**Table 6:** Antiviral agents used or proposed for mpox therapy.

Agent	Mechanism	Route	Regulatory Status (Mpox)
Tecovirimat (ST-246; Tpoxx/ Tecovirimat SIGA)	VP37 (F13L) envelope-protein inhibitor; blocks release of enveloped virions, limiting cell-to-cell spread	Oral (IV for severe disease)	FDA-approved 2018 (smallpox); EMA-authorised 2022 (orthopoxviruses). Following four negative randomised trials, the EMA CHMP recommended in March 2026 that it no longer be used for mpox; other orthopoxvirus indications unaffected [38,45]
Cidofovir	Viral DNA polymerase (E9L) inhibitor	IV	Approved for CMV retinitis; compassionate use only for mpox [2,3]
Brincidofovir (CMX001)	Lipid-conjugated oral pro-drug of cidofovir (E9L)	Oral	FDA-approved for smallpox (animal-efficacy rule); compassionate use for mpox [1,3]
VIGIV	Passive immunisation with orthopoxvirus antibodies	IV	Approved for vaccinia (smallpox-vaccine) complications; compassionate use for mpox [38]

## Data Availability

All data analysed are derived from publicly available sources cited in the manuscript. No primary patient-level data were generated. The review methodology is presented in the Supplementary Materials File.

## Supplementary Materials

The following supporting information can be downloaded at https://www.mdpi.com/article/10.3390/v18080865/s1.
