# Supplementary material for: Mpox in Europe, 2022–2026: A Scoping Review of Viral Clades, Host Determinants of Severity and Antiviral Therapy
Source: Viruses. 2026 Aug 7;18(8):865. doi: 10.3390/v18080865 (PMC13517263; doi:10.3390/v18080865)
Supplement: Supplementary file 1 [file viruses-18-00865-s001.zip › viruses-4452593-supplementary.pdf]

Mpox in Europe, 2022–2026: a scoping review of viral clades, host determinants of severity and antiviral therapy

Supplementary Material

1. PRISMA-ScR compliance statement and protocol

This review was designed, conducted and reported in accordance with the Preferred Reporting Items for Systematic reviews and Meta-Analyses extension for Scoping Reviews (PRISMA-ScR; Tricco AC, Lillie E, Zarin W, et al. Ann Intern Med 2018;169(7):467-473). A completed PRISMA-ScR checklist mapping every one of the 20 items (plus title, funding and structured-summary sub-items) to the location of the corresponding content in the manuscript is provided in Section 2.

A review protocol was drafted internally before commencement of the searches. The protocol was not prospectively registered in PROSPERO because PROSPERO does not accept scoping-review protocols (PROSPERO scope statement, https://www.crd.york.ac.uk/prospero/#aboutregpage). No equivalent registry for scoping reviews was available at the time of protocol development. The internal protocol document is retained by the authors and is available on reasonable request. Deviations from the original protocol are documented in Section 9 (Limitations).

The literature window was 1 January 2022 to 30 April 2026. The final search across all information sources was added on 30 June 2026, updating values where needed. Surveillance and regulatory documents released after 30 April 2026 (for example the ECDC Communicable Disease Threats Report Week 26/2026 and the WHO Situation Report No. 60 of 8 December 2025) were retained as contextual reservoirs and are cited in the text for epidemiological framing only; they were not treated as primary studies.

2. Completed PRISMA-ScR checklist

Every PRISMA-ScR item is presented alongside a signpost to the manuscript section, table or supplementary section in which the corresponding content is reported.

Item	Section / topic	PRISMA-ScR item description (Tricco et al., 2018)	Where reported in this manuscript
**TITLE**			
**1**	Title	Identify the report as a scoping review.	Title page: "...a scoping review of viral clades, host determinants of severity and antiviral therapy".
**ABSTRACT**			
**2**	Structured summary	Provide a structured summary that includes (as applicable): background, objectives, eligibility criteria, sources of evidence, charting methods, results and conclusions that relate to the review questions and objectives.	Abstract (Background / Methods / Results / Conclusions).
**INTRODUCTION**			
**3**	Rationale	Describe the rationale for the review in the context of what is already known. Explain why the review questions/objectives lend themselves to a scoping review approach.	Section 1 (Introduction). See paragraphs on the shift from a Clade IIb to a Clade I/Ib framing, on the narrowing antiviral landscape (EMA March 2026), and on the mismatch between the well-studied mild European experience and the under-studied high-risk groups.
**4**	Objectives	Provide an explicit statement of the questions and objectives being addressed with reference to their key	Section 1 (Introduction), final paragraph: three axes (clades; population demographics; antivirals).

Item	Section / topic	PRISMA-ScR item description (Tricco et al., 2018)	Where reported in this manuscript
		elements (e.g., population or participants, concepts, and context) or other relevant key elements used to conceptualize the review questions and/or objectives.	PCC framework in Supplementary Section 2 below.
**METHODS**			
**5**	Protocol and registration	Indicate whether a review protocol exists; state if and where it can be accessed (e.g., a Web address); and if available, provide registration information, including the registration number.	This review was NOT prospectively registered in PROSPERO because PROSPERO does not accept scoping-review protocols. An internal protocol is retained by the authors and available on reasonable request. See Supplementary Section 1.
**6**	Eligibility criteria	Specify characteristics of the sources of evidence used as eligibility criteria (e.g., years considered, language, and publication status), and provide a rationale.	Supplementary S2 (Population-Concept-Context table). Window: 1 January 2022 – 30 April 2026. Languages: English. Publication status: peer-reviewed articles, reviews, letters, short surveys; conference abstracts excluded unless retrievable in full.
**7**	Information sources*	Describe all information sources in the search (e.g., databases with dates of coverage and contact with authors to identify additional sources), as well as the date the most recent search was executed.	Supplementary Section 3. CCB Central repository (registry), PubMed/ MEDLINE, Scopus (bibliometric map, not for primary inclusion), Google Scholar (Scholar Labs) for backward + forward citation searching. Most recent search: 8 June 2026.
**8**	Search	Present the full electronic search strategy for at least 1 database, including any limits used, such that it could be repeated.	Supplementary Section 4. Full Scopus query reproduced verbatim. PubMed and Google Scholar strategies also given.
**9**	Selection of sources of evidence*	State the process for selecting sources of evidence (i.e., screening and eligibility) included in the scoping review.	Supplementary Section 5. Two-stage screening (title/abstract → full text) against the PCC criteria; dual review with consensus on disagreement.
**10**	Data charting process*	Describe the methods of charting data from the included sources of evidence (e.g., calibrated forms or forms that have been tested by the team before their use, and whether data charting was done independently or in duplicate) and any processes for obtaining and confirming data from investigators.	Supplementary Section 6. Structured charting form with 14 pre-specified variables; piloted on 10 records; independent charting by two reviewers with consensus arbitration; no author contact required (all data extractable from published sources).
**11**	Data items	List and define all variables for which data were sought and any assumptions and simplifications made.	Supplementary Section 6. 14 items: study ID, CCB link, author/year, country, design, sample size, population type, clade/lineage, exposure/predictor, HIV status, CD4, immunosuppression, outcome (CFR / hospitalisation / severity / immunogenicity), and effect size with 95 % CI.
**12**	Critical appraisal of individual sources of evidence†	If done, provide a rationale for conducting a critical appraisal of included sources of evidence; describe the methods used and how	Formal risk-of-bias appraisal was NOT undertaken, which is appropriate for a PRISMA-ScR scoping review (Tricco et al., 2018). Study-design and setting

Item	Section / topic	PRISMA-ScR item description (Tricco et al., 2018)	Where reported in this manuscript
		this information was used in any data synthesis (if appropriate).	information were retained in the charting table to inform narrative interpretation.
**13**	Synthesis of results	Describe the methods of handling and summarizing the data that were charted.	Section 3 (Results) of the manuscript + Supplementary Section 7. Narrative and tabular synthesis by the three axes; a Scopus bibliometric snapshot; no pooled meta-analytic estimate was computed because of design heterogeneity; results from included evidence syntheses were cited, not re-pooled.
**RESULTS**			
**14**	Selection of sources of evidence	Give numbers of sources of evidence screened, assessed for eligibility, and included in the review, with reasons for exclusions at each stage, ideally using a flow diagram.	Figure 1 (PRISMA-ScR flow diagram). Records identified by arm; de-duplication; title/abstract screening; full-text assessment; final inclusion.
**15**	Characteristics of sources of evidence	For each source of evidence, present characteristics for which data were charted and provide the citations.	Supplementary Table S3 (cohort inventory) + main-text Table 2 (per-country notes) + Table 3 (clade-stratified outcomes) + Table 4 (severity predictors). Manuscript references S01-S39 plus cohort keys.
**16**	Critical appraisal within sources of evidence	If done, present data on critical appraisal of included sources of evidence (see item 12).	Not applicable (see item 12).
**17**	Results of individual sources of evidence	For each included source of evidence, present the relevant data that were charted that relate to the review questions and objectives.	Section 3.1 (clades / geography) + Section 3.2 (demographic and immunological predictors) + Section 3.3 (antivirals). Supplementary Table S3 additionally lists cohort-level extractions.
**18**	Synthesis of results	Summarize and/or present the charting results as they relate to the review questions and objectives.	Manuscript Sections 3 and 4; visualised in Figures 3-8 and Table 4.
**DISCUSSION**			
**19**	Summary of evidence	Summarize the main results (including an overview of concepts, themes, and types of evidence available), link to the review questions and objectives, and consider the relevance to key groups.	Section 4 (Evidence gaps and research priorities) + Section 5 (Conclusions).
**20**	Limitations	Discuss the limitations of the scoping review process.	Supplementary Section 9 below (and briefly noted in the Discussion of the manuscript).
**21**	Conclusions	Provide a general interpretation of the results with respect to the review questions and objectives, as well as potential implications and/or next steps.	Section 5 (Conclusions) of the manuscript.
**FUNDING**			

Item	Section / topic	PRISMA-ScR item description (Tricco et al., 2018)	Where reported in this manuscript
**22**	Funding	Describe sources of funding for the included sources of evidence, as well as sources of funding for the scoping review. Describe the role of the funders of the scoping review.	Declared in the manuscript ("Funding" statement): no specific grant received. Funding of included sources of evidence: reported by original authors; not systematically re-extracted.

** Sources of evidence: information sources; selection of sources of evidence; and data charting process are subject to editorial adaptation from the original PRISMA 2009 checklist for scoping reviews. † Critical appraisal is optional in a scoping review (PRISMA-ScR item 12).*

3. Eligibility criteria (Population – Concept – Context)

Eligibility followed the PCC framework recommended by the Joanna Briggs Institute for scoping reviews (Peters et al., JBI Manual for Evidence Synthesis, Chapter 11, 2020).

Element	Inclusion criteria	Exclusion criteria
**Population**	Humans with, or at risk of, mpox; vaccinated populations. European (EU/EEA + UK) focus; global and African data included where European evidence is sparse (Clade I/Ib severity, paediatric burden).	Animal-only studies without clinical or translational relevance.
**Concept**	Viral clade and lineage taxonomy; demographic and immunological predictors of outcome; antiviral safety, efficacy and resistance; evidence gaps.	Records with no extractable clade, outcome or antiviral variable.
**Context**	European clinical and public-health practice, 2022–2026; African/global comparators used only for Clade I severity and paediatric burden where European data are absent.	Non-mpox orthopoxvirus disease only (smallpox vaccination complications, cowpox).
**Types of source**	CCB cohort-project outputs, prospective and retrospective cohort studies, cross-sectional studies, case-control studies, case series and reports, randomised trials, systematic and narrative reviews, meta-analyses, surveillance bulletins, regulatory guidance (ECDC, WHO, UKHSA, EMA).	Duplicate cohort records (least complete removed); conference abstracts without a retrievable full text or extractable data.
**Time**	Records dated 1 January 2022 – 30 April 2026 (publication or reporting date).	Records published outside the window unless they constitute a citing/cited link to an in-window record.
**Language**	English.	Non-English records not translated.

4. Information sources

Source	Role	Coverage / date of last search	Records retrieved
**CCB Central Data Repository (cohortcoordinationboard.eu/central-data-repository)**	Primary source (registry)	1 Jan 2022 - 31 May 2026	47 mpox-related European cohort projects identified; 21 publications and outputs retrieved and screened. Forms the principal cohort evidence base of the review.
**PubMed / MEDLINE**	Primary source (bibliographic database)	1 Jan 2022 - 8 Jun 2026 (search executed 8 Jun 2026)	Search restricted to evidence-synthesis and trial article types (Meta-Analysis, Randomized Controlled Trial, Review, Systematic Review). Records identified: n = 12.

Source	Role	Coverage / date of last search	Records retrieved
**Scopus**	Bibliometric map (not a primary inclusion source)	1 Jan 2022 - 30 Apr 2026 (search executed 8 Jun 2026)	1,271 records. Used to characterise the publication landscape (annual output, dominant title terms, thematic distribution) (see Figure 1 of the manuscript). No records were added to the included set from Scopus alone.
**Google Scholar (Scholar Labs)**	Supplementary source (backward + forward citation searching)	1 Jan 2022 - 8 Jun 2026	39 mpox-related papers retrieved to capture recent or not-yet-indexed work.
**ECDC, UKHSA, WHO, EMA — surveillance and regulatory grey literature**	Contextual reservoir (not screened as primary studies)	Documents released 2022 - Jun 2026 (Communicable Disease Threats Report Week 26/2026; WHO Situation Reports up to No. 60, 8 Dec 2025; UKHSA 2026 briefing; EMA CHMP March 2026 opinion; ECDC Threat Assessment Brief 24 Oct 2025)	Used for epidemiological framing and dates only; every quantitative claim taken from these documents is cited in the text.

The CCB Central Data Repository was treated as the backbone of the review because it is the only pan-European registry that consolidates cohort projects from national teams under a common metadata schema. PubMed provided coverage of peer-reviewed systematic reviews, meta-analyses and randomised trials. Google Scholar was used only for backward and forward citation chasing from records already screened. Scopus was used as a bibliometric map of the wider field (annual publication output, dominant title terms, thematic distribution) and did not contribute records to the included set independently of the other arms.

5. Search strategies

5.1 CCB Central Data Repository (registry)

The CCB repository was filtered by disease term ("mpox", "monkeypox", "MPXV") and by European scope. All projects returned by the filter were retained; project pages were followed to their publication outputs (peer-reviewed articles, consortium reports and pre-prints).

5.2 PubMed / MEDLINE

The PubMed strategy combined disease and geography terms and was restricted to evidence-synthesis and trial article types:

```
(mpox OR monkeypox OR "monkeypox virus" OR MPXV OR Mpox) AND (Europe OR EU OR EEA)
```

Filters applied: Article type = Meta-Analysis OR Randomized Controlled Trial OR Review OR Systematic Review. Language = English. Publication date = 2022-01-01 to 2026-04-30. Search executed 8 June 2026. Records identified from this arm: n = 12 (export file: abstract_mpoxtiabOR_set.txt).

5.3 Scopus (bibliometric map)

The Scopus query, reproduced verbatim from the search log:

```
( TITLE-ABS-KEY ( mpox OR monkeypox OR "monkeypox virus" OR mpxv ) ) AND ( TITLE-ABS-KEY ( clade OR
lineage OR phylogen* OR sever* OR hospitali*   OR mortalit* OR "case fatality" OR HIV OR CD4 OR immunosupp* OR
pregnan*   OR p?ediatric OR demograph* OR predictor* OR tecovirimat OR cidofovir   OR brincidofovir OR vigiv OR
antiviral* OR resistance ) ) AND ( AFFILCOUNTRY ( austria OR belgium OR bulgaria OR croatia OR cyprus   OR "czech
```

```
republic" OR denmark OR estonia OR finland OR france OR germany   OR greece OR hungary OR iceland OR ireland
OR italy OR latvia   OR liechtenstein OR lithuania OR luxembourg OR malta OR netherlands   OR norway OR poland
OR portugal OR romania OR slovakia OR slovenia   OR spain OR sweden OR "united kingdom" ) ) AND PUBYEAR >
2021 AND PUBYEAR < 2027 AND ( LIMIT-TO ( LANGUAGE , "English" ) ) AND ( LIMIT-TO ( DOCTYPE , "ar" ) OR
LIMIT-TO ( DOCTYPE , "re" )   OR LIMIT-TO ( DOCTYPE , "le" ) OR LIMIT-TO ( DOCTYPE , "sh" ) )
```

Records returned: 1,271 (search executed 8 June 2026). Used only as a bibliometric map; no records were added to the included set from Scopus alone.

5.4 Google Scholar (Scholar Labs)

Used for backward and forward citation chasing from records already screened in the CCB and PubMed arms. 39 papers were retrieved and taken forward to title/abstract screening.

6. Selection of sources of evidence

Records from all arms were exported and de-duplicated by Digital Object Identifier (DOI). A three-stage selection procedure followed.

- Stage 1 (title / abstract screening): two reviewers screened records independently against the PCC criteria; disagreements were resolved by consensus.
- Stage 2 (full-text retrieval and eligibility assessment): potentially eligible reports were retrieved; the same two reviewers independently assessed each for final eligibility; disagreements were again resolved by consensus.
- Stage 3 (charting): the included set was charted into the pre-specified 14-item form (Section S7).

Reasons for full-text exclusion were logged for every excluded record and are summarised in Figure 1 in the main text. The final included set contains 80 records (as listed in the manuscript's Bibliography.bib), corresponding to the reference keys S01–S39 plus the cohort-author keys (MOSAIC, ICONA, Mpox_Cohort, GermanMpox, POQS, ITM2, ITM3, Svartstein, Packer, Kowalski, MoVIHvax_author, Fontan_Vela, MArtinez_arias, STD_MAR, Moschese, Nozza, Catalano, Clemente, Ambrosioni2026, Vasudevan2025, Kassaw2026, Rubini2026, Akhavan2025, Pastornacki2025, Baker2026, PALM007, STOMP, UNITY, PLATINUMUK, EMA2026) and the surveillance / regulatory keys (ECDC2026a, ECDC2026b, ECDC2026c, ECDC2025TAB, UKHSA2026, WHO2025a, WHO2025b, WHO_SR60, PRISMA_ScR, CCBCentral2022).

7. Data charting form

A single charting form with 14 pre-specified variables was piloted on 10 records (5 cohort studies and 5 reviews) before being applied to the full included set. Charting was performed independently by two reviewers with consensus arbitration. No primary authors were contacted, since every variable of interest could be extracted from published sources.

#	Variable	Definition / values
1	**Study identifier**	Manuscript reference key and, where present, the CCB project identifier.
2	**First author, year**	Corresponding author of the record and year of publication.
3	**Country / region**	Country of data collection; for multinational cohorts, the list of participating countries.
4	**Study design**	Cohort (prospective / retrospective) / cross-sectional / case-control / case series / case report / RCT / systematic review / narrative review / surveillance bulletin / regulatory document.
5	**Sample size**	Total sample size and, where relevant, sub-sample sizes (e.g., PWH, MSM, paediatric, pregnant).
6	**Population type**	MSM, people with HIV (PWH), STI-clinic attendees, household contacts, paediatric (<18 y), pregnant, older adults (≥65 y), mixed.
7	**Clade / lineage**	Clade I / Ia / Ib / II / IIa / IIb; lineage where reported (B.1, C.1, A.1).
8	**Exposures / predictors**	HIV status, CD4 count, non-HIV immunosuppression, age, sex, pregnancy, prior STI, vaccination status.
9	**Case fatality rate (CFR)**	Deaths / cases; numerator, denominator, timeframe.
10	**Hospitalisation rate**	Hospitalised / cases; setting; length of stay if reported.

#	Variable	Definition / values
11	**Severity indicators**	Extensive lesions, mucosal / perianal complications, bacterial superinfection, ICU admission.
12	**Viral shedding / virology**	Viral load, PCR positivity by site, time-to-clearance.
13	**Antiviral use and outcomes**	Tecovirimat / cidofovir / brincidofovir / VIGIV. Indication, dose, duration, outcome, adverse events, resistance.
14	**Effect estimate + 95 % CI**	As reported by the primary source, not re-computed.

8. Synthesis methods

Synthesis was narrative and tabular, structured by the three thematic axes of the review (clades and geography, demographic and immunological host determinants of severity, antiviral therapy and resistance) and supported by a study/characteristics mapping of the included cohort evidence (Section 10). A bibliometric snapshot of the wider field was derived from the Scopus corpus and is presented in Figure 1 of the manuscript.

Because the included evidence spans heterogeneous designs (surveillance bulletins, prospective cohorts, retrospective cohorts, randomised trials, case series and regulatory documents), no single pooled effect estimate was computed. Estimates from included evidence syntheses (Vasudevan 2025; Kassaw 2026) were cited, not re-pooled. Epidemiological context was drawn from ECDC (2026a, 2026b, 2026c), UKHSA (2026), WHO (2025a, 2025b) and WHO Situation Report No. 60 (2025); EU/EEA case counts referred to in the manuscript are computed from the WHO global mpox dataset (cumulative data to 30 April 2026).

9. Evidence-gap scoring rubric

21 evidence gaps identified during charting were scored on five dimensions by two reviewers with consensus arbitration; the composite score (range 5-15) was mapped to the four ordinal priority tiers used in the priority heatmap (Figure 8 of the manuscript). This scoring is a structured summary of reviewer judgement, not a measured value. The rubric is reproduced below for transparency.

Dimension (score 1–3)	Score 1	Score 2	Score 3
**Clinical / public-health impact**	Minor	Affects some patients	Bears on severe outcomes or mortality
**Size of the evidence deficit**	Direct European data exist	Only indirect / extrapolated data	No European data
**Population vulnerability**	General population	Moderate-risk subgroup	Highest-risk (advanced HIV, non-HIV immunosuppression, paediatric, pregnant)
**Timeliness / emerging relevance**	Stable question	Relevant to practice	Urgent given Clade I emergence, resistance or regulatory change
**Actionability / feasibility**	Hard to address	Moderate effort	Readily addressable with a defined study

Composite score (sum, range 5–15)	Priority tier (used in Figure 8)
5–7	**Future priority**
8–10	**Moderate**
11–12	**High**
13–15	**High – urgent**

10. European cohort projects in this review

Table S3 lists the European cohort projects and programmes that constitute the principal evidence base of the review. Several are coordinated through the CCB Central Data Repository. The list is representative and is not exhaustive. Every cohort included has been surfaced from the CCB filter, the PubMed evidence-synthesis filter, or backward/forward citation chasing.

European cohort / programme	Country / region	Design (n)
**MOSAIC observational cohort**	6 EU countries	Multicentre observational (Clade IIb)
**mpox-ICONA**	Italy	Prospective multicentre (n = 541)
**PISCIS**	Spain (Catalonia)	Cohort (n = 673; CD4 strata)
**ITM Mpox cohort / POQS**	Belgium	Prospective + retrospective (n = 186 – 447)
**German multicentre cohort**	Germany	Large outbreak cohort (men, HIV +/–)
**Svartstein MSM-HIV cohort**	Denmark	Longitudinal (n = 727)
**UK household-contact study**	United Kingdom	Contact study (n = 1,526 contacts)
**Primary-care detection programme**	Spain	Case-finding outside sexual-health services
**Hospital cohort (Kowalski)**	Poland	HIV+/– comparison (median 31 y)
**ITM3 / WHO European Region**	EU / EEA	Clade I epidemiology, 2024–2025
**MoVIHvax / STD-MAR**	Spain	Vaccine effectiveness (JYNNEOS)
**Moschese / Nozza / Catalano / Clemente**	Italy	Regional cohort and case series
**French national cohort**	France	Paris metropolitan cluster (n unspecified)
**EACS guidelines v13.0 (contextual)**	EU-wide	Guideline document
**Project ODIN (contextual)**	EU + Africa	Consortium report on genomic surveillance

11. Limitations of the review process

- The CCB-led inclusion strategy captures European cohort outputs but may under-represent non-cohort or non-CCB-affiliated work. This is mitigated by the PubMed evidence-synthesis arm and by the Google Scholar citation-chasing arm.
- The Scopus corpus served as a bibliometric map of the field, not as a primary inclusion source, so records indexed only in Scopus were not automatically included.
- The near-monoclonal Clade IIb composition of the European case population during 2022–2024 precludes intra-European clade-severity comparison; Clade I/Ib severity estimates rely on African surveillance and early European detections. This is discussed at length in Sections 3.1 and 4 of the manuscript.
- Formal risk-of-bias appraisal was not conducted, which is standard for a PRISMA-ScR scoping review; readers should interpret narrative conclusions accordingly.
- Non-English sources were not translated. This may under-represent Central and Eastern European experience.
- The literature window closed on 30 April 2026. Subsequent surveillance and regulatory releases (ECDC Communicable Disease Threats Report Week 26/2026 covering June 2025 - May 2026; WHO Situation Report No. 60 of 8 December 2025) are cited only for epidemiological framing and did not undergo the same screening process as primary studies.

12. Data availability

All data analysed in this review are derived from publicly available sources cited in the manuscript. No primary patient-level data were generated by the authors. The Scopus export used for the bibliometric analysis, the PubMed export, and the internal protocol document are retained by the authors and are available on reasonable request. The PRISMA-ScR checklist (Section 2), the charting form (Section 7) and the evidence-

gap scoring rubric (Section 9) are provided in full above, while the completed flow diagram is given in the main text (Fig. 1).

13. References

- Tricco AC, Lillie E, Zarin W, et al. PRISMA extension for scoping reviews (PRISMA-ScR): checklist and explanation. Ann Intern Med. 2018;169(7):467–473. doi:10.7326/M18-0850
- Peters MDJ, Godfrey C, McInerney P, Munn Z, Tricco AC, Khalil H. Chapter 11: Scoping reviews. In: Aromataris E, Munn Z, editors. JBI Manual for Evidence Synthesis. Adelaide: JBI; 2020.
- Cohort Coordination Board (CCB) Central Data Repository. https://cohortcoordinationboard.eu/central-data-repository/ [accessed 8 June 2026].
- PROSPERO — International prospective register of systematic reviews. Scope statement: https://www.crd.york.ac.uk/prospero/#aboutregpage
